# USP26 Combats Age‐Related Declines in Self‐Renewal and Multipotent Differentiation of BMSC by Maintaining Mitochondrial Homeostasis

**DOI:** 10.1002/advs.202406428

**Published:** 2024-10-08

**Authors:** Yiming Xu, Leilei Chang, Yong Chen, Zhou Dan, Li Zhou, Jiyuan Tang, Lianfu Deng, Guoqing Tang, Changwei Li

**Affiliations:** ^1^ Department of Orthopedics Shanghai Key Laboratory for Prevention and Treatment of Bone and Joint Diseases Shanghai Institute of Traumatology and Orthopedics Ruijin Hospital Shanghai Jiao Tong University School of Medicine 197 Ruijin 2nd Road Shanghai 200025 China; ^2^ Department of Orthopedics Kunshan Hospital of Chinese Medicine Affiliated Hospital of Yangzhou University Suzhou Jiangsu Province 215300 China; ^3^ Institute of Traumatology and Orthopedics Kunshan Hospital of Chinese Medicine Affiliated Hospital of Yangzhou University Suzhou Jiangsu Province 215300 China

**Keywords:** aging, BMSC, mitochondrial homeostasis, multipotency, self‐renewal, USP26

## Abstract

Age‐related declines in self‐renewal and multipotency of bone marrow mesenchymal stem cells (BMSCs) limit their applications in tissue engineering and clinical therapy. Thus, understanding the mechanisms behind BMSC senescence is crucial for maintaining the rejuvenation and multipotent differentiation capabilities of BMSCs. This study reveals that impaired USP26 expression in BMSCs leads to mitochondrial dysfunction, ultimately resulting in aging and age‐related declines in the self‐renewal and multipotency of BMSCs. Specifically, decreased USP26 expression results in decreased protein levels of Sirtuin 2 due to its ubiquitination degradation, which leads to mitochondrial dysfunction in BMSCs and ultimately resulting in aging and age‐related declines in self‐renewal and multilineage differentiation potentials. Additionally, decreased USP26 expression in aging BMSCs is a result of dampened hypoxia‐inducible factor 1α (HIF‐1α) expression. HIF‐1α facilitates USP26 transcriptional expression by increasing USP26 promoter activity through binding to the ‐191 — ‐198 bp and ‐262 — ‐269 bp regions on the USP26 promoter. Therefore, the identification of USP26 as being correlated with aging and age‐related declines in self‐renewal and multipotency of BMSCs, along with understanding its expression and action mechanisms, suggests that USP26 represents a novel therapeutic target for combating aging and age‐related declines in the self‐renewal and multipotent differentiation of BMSCs.

## Graphical abstract

This work shows the schematic of how USP26 combats age‐related declines in self‐renewal and multipotent differentiation of BMSC by maintaining mitochondrial homeostasis. As bone marrow mesenchymal stem cells (BMSCs) age, decreased hypoxia‐inducible factor 1α (HIF‐1α) expression reduces its binding to the ‐191 — ‐198 and ‐262 — ‐269 bp regions on the USP26 promoter, thereby dampening Usp26 transcriptional expression. This leads to reduced protein expression of SIRT2 through the promotion of its ubiquitination and degradation. Consequently, mitochondrial dysfunction occurs in BMSCs, resulting in senescence phenotypes characterized by decreased self‐renewal and impaired multilineage differentiation potentials.

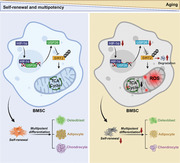

## 1 Introduction

Mesenchymal stem cells (MSCs), which originate from the mesoderm, are stem cells with the ability to self‐renewal while maintaining multipotent differentiation potential.^[^ ^1^ ^]^ They can be induced to differentiate into various tissues, including osteoblasts, adipocytes, and chondrocytes under specific conditions.^[^ ^2^ ^]^ This unique property of MSCs presents significant potential applications in tissue engineering and clinical therapy, particularly in cell replacement therapy, gene therapy, and tissue and organ regeneration.^[^ ^3^ ^]^ However, as indicated by numerous studies, MSCs undergo functional decline and gradually lose their multipotency with age or prolonged culturing, which limits their therapeutic use.^[^ ^4^ ^]^ Therefore, understanding the possible mechanisms underlying MSC senescence is crucial for promoting and maintaining the self‐renewal and multipotent differentiation capabilities of aging MSCs.

Deubiquitination is crucial for the lineage differentiation of bone marrow mesenchymal stem cells (BMSCs).^[^ ^5^ ^]^ Deubiquitinating enzymes (DUBs) can remove ubiquitin molecules from protein substrates to maintain their stability.^[^ ^6^ ^]^ The ubiquitin‐specific protease (USP) family is the largest among the five families of DUBs, comprising more than 50 known members.^[^ ^7^ ^]^ Recent studies also suggest that the USP family plays a vital role in regulating cell stemness and multipotency.^[^ ^8^ ^]^ In our previous research, we found that USP26 acts as a regulator of bone homeostasis by coordinating bone formation and resorption.^[^ ^9^ ^]^ Additionally, we discovered that decreased expression of USP26 is a major cause of impaired osteogenic and chondrogenic differentiation in aged BMSCs, indicating that USP26 may be involved in aging and multilineage differentiation declines of BMSCs.

This study has found that decreased USP26 expression is correlated with aging, reduced self‐renewal ability, and impaired multipotent differentiation potentials of BMSCs. Mechanistically, the impairment of the hypoxia‐inducible factor 1α (HIF‐1α) pathway in aging BMSCs leads to reduced transcriptional expression of Usp26, resulting in decreased protein expression of SIRT2 due to the promotion of SIRT2 ubiquitination and degradation. This ultimately leads to mitochondrial dysfunction in BMSCs, resulting in senescence phenotypes, such as decreased proliferation and impaired multilineage differentiation potentials. Therefore, the identification of USP26 as being correlated with aging and age‐related declines in the self‐renewal and multipotency of BMSCs, along with understanding its expression and action mechanisms, suggests that USP26 could represent a novel therapeutic target for combating aging and age‐related declines in the self‐renewal and multipotent differentiation of BMSCs.

## 2 Results

### 2.1 Decreased USP26 Expression is Correlated with the Aging of BMSCs

**Figure 1:**
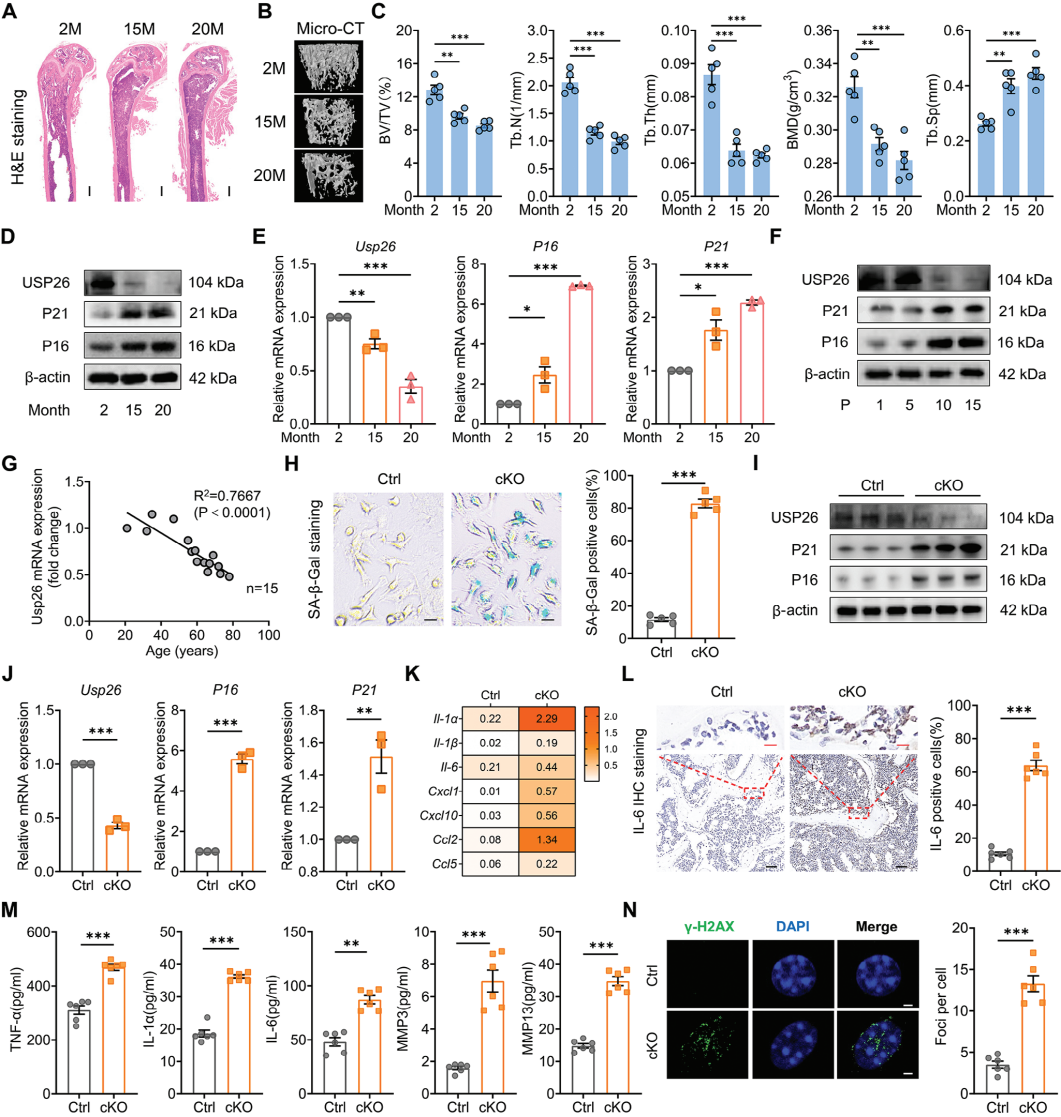
The decreased USP26 expression is correlated with aging of BMSCs. A) Representative H&E staining images of the femurs from 2‐month‐old, 15‐month‐old, and 20‐month‐old mice. n = 5 in each group. Scale bar, 500 µm. B) Representative Micro‐CT images of the femurs from 2‐month‐old, 15‐month‐old, and 20‐month‐old mice. n = 5 in each group. C) Quantitative analysis of the trabecular bone from 2‐month‐old, 15‐month‐old, and 20‐month‐old mice including BV/TV, Tb.N, Tb.Th, Tb.Sp, and BMD. n = 5 in each group. D) Western‐blot analysis of USP26, P21, and P16 protein levels in BMSCs from mice of different ages (2, 15, and 20 months). n = 3 in each group. E) qPCR analysis of Usp26, P16, and P21 mRNA expressions in BMSCs from mice of different ages (2, 15, and 20 months). n = 3 in each group. F) Western blot analysis of USP26, P21, and P16 protein levels in BMSCs of different generations (1, 5, 10, and 15). n = 3 in each group. G) qPCR analysis of Usp26 mRNA expressions from the bone marrow of different patients (n = 15) and evaluation of the relevance between Usp26 mRNA expression and age. H) Representative SA‐β‐Gal staining images and percentage of SA‐β‐Gal positive cells of BMSCs from control and cKO mice. n = 5 in each group. Scale bar, 10 µm. I) Western blot analysis of USP26, P21, and P16 protein levels of BMSCs from control and cKO mice. n = 3 in each group. J) qPCR analysis of Usp26, P16, and P21 mRNA expressions of BMSCs from control and cKO mice. n = 3 in each group. K) qPCR analysis of SASP‐related gene (Il‐1α, Il‐1β, Il‐6, Cxcl1, Cxcl10, Ccl2, and Ccl5) mRNA expressions of BMSCs from control and cKO mice. n = 3 in each group. L) Representative Immunohistochemistry (IHC) staining of IL‐6 of the femurs from control and cKO mice. IL‐6 positive cells number as a quantitative measurement. n = 6 in each group. Scale bar, 50 µm (black) and 10 µm (red). M) Total protein lysates of bone marrow supernatant from the femurs of control and cKO mice were analyzed for TNF‐α, IL‐1α, IL‐6, MMP3, and MMP13. n = 6 in each group. N) Representative immunofluorescent images of γ‐H2AX foci in BMSCs from control and cKO mice and quantification of the number of γ‐H2AX foci per cell. n = 6 in each group. Mice age in H), I), J), K), L), M), and N), 2‐month‐old. BMSCs from mice were used in D), E), F), H), I), J), K), M), and N); BMSCs from Human were used in G). Data are represented as mean ± SD. Statistical significance was determined by one‐way ANOVA in C) and E), and two‐sided student's t test in H), J), L), M) and N). *p < 0.05, **p < 0.01, ***p < 0.001.

To investigate the role of USP26 in the aging of BMSCs, we collected BMSCs from the long bones of mice aged 2 months, 15 months, and 20 months of age. **Figure** **1**A–E demonstrates that as mice aged and exhibited senile bone loss, BMSCs showed increased expression of aging‐related markers such as P16 and P21. In contrast, the expression of USP26 gradually decreased. This negative correlation was confirmed in in vitro cultures of BMSCs that were expanded for 15 passages (Figure 1F). In addition, an age‐dependent decrease in mRNA expression of USP26 was further confirmed in human BMSCs extracted from various age groups (Figure 1G). These findings suggest that the decreased expression of USP26 may be involved in the aging process of BMSCs.

To investigate the effects of USP26 deletion on the aging process of BMSCs, BMSCs were obtained from Usp26 cKO (Usp26^flox/flox^; Prx1‐Cre) mice and their wild type (WT) littermates (Usp26^flox/flox^). Paired related homeobox (Prx1) is a transcriptional coactivator, which is expressed during limb bud development, and Prx1‐Cre is widely used to delete genes in multipotent mesenchymal cells, including BMSCs.^[^ ^10^ ^]^ Thus, herein we conditionally deleted Usp26 in BMSCs using Prx1‐Cre. The Usp26 cKO mice were viable and born at the expected Mendelian ratio, and their body sizes and weights were comparable to those of the 2‐month‐old WT littermates. Compared to those of the WT controls, BMSCs isolated from Usp26 cKO mice displayed an increased number of β‐galactosidase‐positive cells, indicating a higher level of cellular aging (Figure 1H). Furthermore, the protein and mRNA levels of the aging markers P21 and P16 were notably elevated in BMSCs derived from Usp26 cKO mice (Figure 1I,J). The upregulation of P21 and P16 resulted in a stable cell cycle arrest, accumulation of cellular damage, and impaired repair mechanisms, leading to premature aging. This process is often accompanied by the senescence‐associated secretory phenotype (SASP).^[^ ^11^ ^]^ In cells derived from Usp26 cKO mice, there was a marked increase in SASP‐related genes, including Il‐1α, Il‐1β, Il‐6, Cxcl1, Cxcl10, Ccl2, and Ccl5 (Figure 1K). Immunohistochemistry (IHC) results of the femur of Usp26 cKO mice also revealed a significant increase in IL‐6‐positive cells (Figure 1L); ELISA results further confirmed that Usp26 cKO BMSCs secreted higher levels of SASP‐related factors and proteins, such as TNF‐α, IL‐1α, IL‐6, MMP3, and MMP13 (Figure 1M). Additionally, BMSCs derived from Usp26 cKO mice exhibited a greater number of γ‐H2AX foci compared to the control, further indicating that the loss of Usp26 resulted in DNA damage and accelerated cell aging (Figure 1N). In conclusion, these findings suggest that reduced USP26 expression is closely linked to the aging of BMSCs.

### 2.2 Decrease USP26 Expression Results in the Self‐Renewal and Multipotency Declines of BMSCs

**Figure 2:**
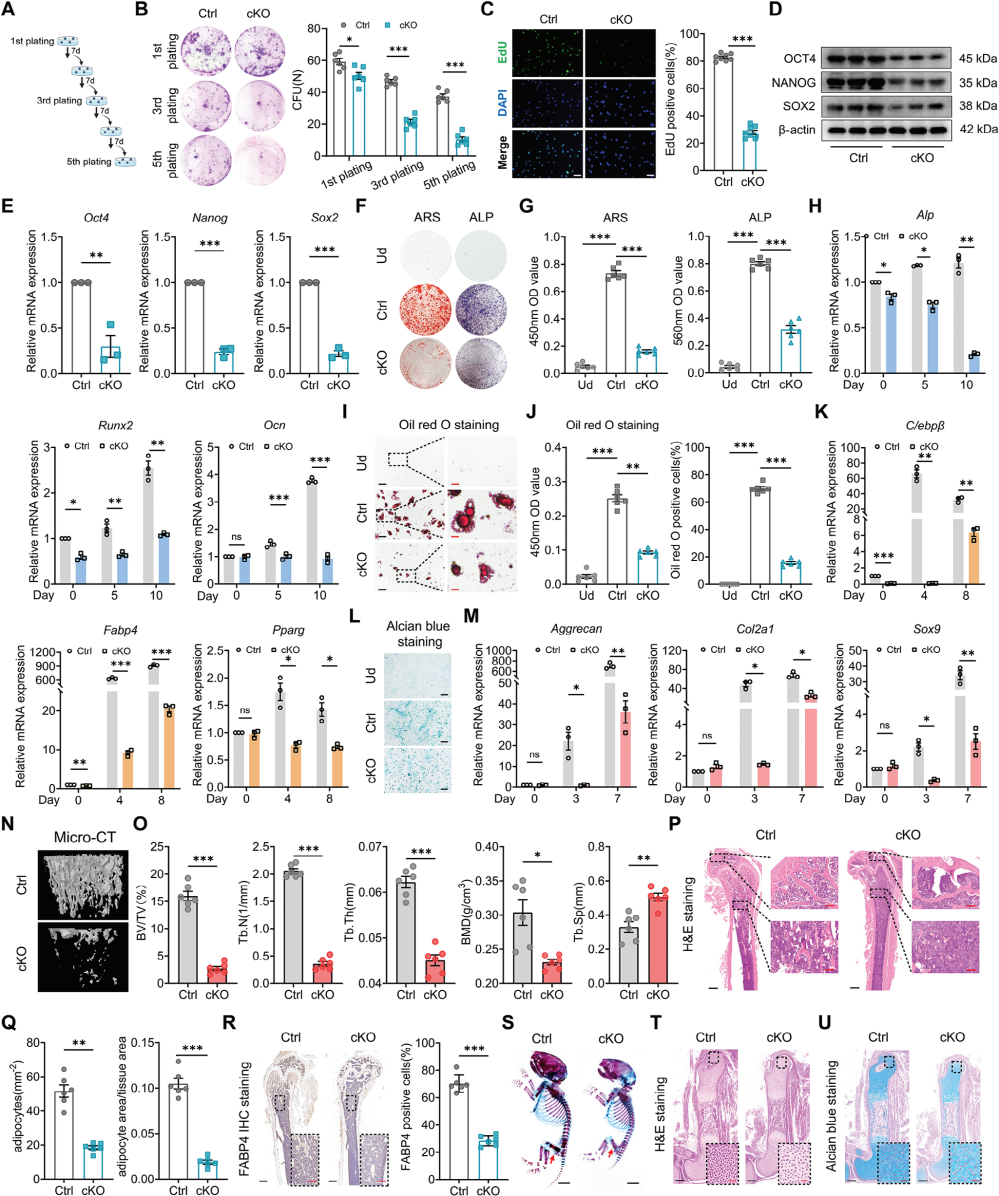
The decrease of USP26 resulted in self‐renewal and multipotency declines of BMSCs in vitro and in vivo. A) Schematic diagram of the serial colony formation unit (CFU) assay through re‐plating. B) Representative images of CFU assay of Usp26 cKO BMSCs and their WT controls after the indicated number of serial re‐plating. Colony number as a quantitative measurement. n = 6 in each group. C) Representative EdU staining images and percentage of EdU positive cells in BMSCs from control and cKO mice. n = 8 in each group. Scale bar, 20 µm. D) Western‐blot analysis of OCT4, NANOG, and SOX2 protein levels in BMSCs from control and cKO mice. n = 3 in each group. E) qPCR analysis of Oct4, Sox2, and Nanog mRNA expressions in BMSCs from control and cKO mice. n = 3 in each group. F) Representative images of ARS and ALP of BMSCs from control and cKO mice after 14 days of osteogenic induction. n = 6 in each group. G) Statistical analysis of the absorbance at 450 nm after ARS and the absorbance at 560 nm after ALP staining. n = 6 in each group. H) qPCR analysis of Alp, Runx2, and Ocn mRNA expressions in BMSCs from control and cKO mice after different days (0, 5, and 10 days) of osteogenic induction. n = 3 in each group. I) Representative images of Oil red O staining of BMSCs from control and cKO mice after 21 days of adipogenic induction. n = 6 in each group. Scale bar, 20 µm (black) and 5 µm (red). J) Statistical analysis of the absorbance at 450 nm after Oil red O staining and percentage of Oil red O positive cells in BMSCs from control and cKO mice. n = 6 in each group. K) qPCR analysis of C/ebpβ, Fabp4, and Pparg mRNA expressions in BMSCs from control and cKO mice after different days (0, 4, and 8 days) of adipogenic induction. n = 3 in each group. L) Representative images of Alcian blue staining of BMSCs from control and cKO mice after 7 days of chondrogenic induction. n = 6 in each group. Scale bar, 50 µm. M) qPCR analysis of Aggrecan, Col2a1, and Sox9 mRNA expressions in BMSCs from control and cKO mice after different days (0, 3, and 7 days) of chondrogenic induction. n = 3 in each group. N) Representative Micro‐CT images of the femurs from control and cKO mice. n = 6 in each group. O) Quantitative analysis of the trabecular bone from control and cKO mice including BV/TV, Tb.N, Tb.Th, Tb.Sp, and BMD. n = 6 in each group. P) Representative H&E staining images of the femurs from control and cKO mice. n = 6 in each group. Scale bar, 500 µm (black) and 100 µm (red). Q) Adipocyte number per tissue area and area of adipocytes per tissue area were measured based on H&E staining images. n = 6 in each group. R) Representative IHC staining of FABP4 of the femurs from control and cKO mice. FABP4 positive cells number serves as a quantitative measurement. n = 6 in each group. Scale bar, 500 µm (black) and 200 µm (red). S) Representative images of the whole skeleton of control and cKO embryos at E16.5. Red arrows indicate delayed alizarin red staining in the femurs. n = 6 in each group. Scale bar, 2.5 mm. T) Representative H&E staining images of the femurs from control and cKO embryos at E16.5. n = 6 in each group. Scale bar, 150 µm (black) and 50 µm (red). U) Representative images of Alcian blue staining of the femurs from control and cKO embryos at E16.5. n = 6 in each group. Scale bar, 150 µm (black) and 50 µm (red). Mice age in B) to R), 2‐month‐old. Mice age in S) to U), E16.5. BMSCs from mice at P5 were used in C)‐M). Data are represented as mean ± SD. Statistical significance was determined by two‐sided Student's t test in C), E), O), Q) and R), one‐way ANOVA in G), and J), and two‐way ANOVA in B), H), K), and M). *p < 0.05, **p < 0.01, ***p < 0.001.

Self‐renewal and multipotent differentiation potential are two important characteristics of BMSCs,^[^ ^12^ ^]^ which have the ability to self‐renew and differentiate into osteoblasts, adipocytes, and chondrocytes in bone tissue.^[^ ^2a–c^ ^]^ BMSC senescence can lead to impaired differentiation potential and a decline in proliferation ability.^[^ ^13^ ^]^ Therefore, after observing that decreased USP26 expression is correlated with the aging of BMSCs, we aimed to investigate whether the decrease of USP26 expression leads to a decline in the self‐renewal and multilineage differentiation potentials of BMSCs. Results from the indicated plating of serial colony‐forming unit (CFU) assay^[^ ^14^ ^]^ showed impaired self‐renewal in BMSCs derived from Usp26 cKO mice (**Figure** **2**A,B). Similarly, EdU staining also showed a significant decrease in proliferation ability of Usp26 cKO BMSCs (Figure 2C). Furthermore, the multipotency of BMSCs is regulated by a series of transcription factors, including NANOG, OCT4, and SOX2. We also observed a significant decrease in the expression of NANOG, OCT4, and SOX2 in the Usp26 cKO BMSCs at both the protein and mRNA levels (Figure 2D,E).

Next, we assessed the trilineage differentiation ability of BMSCs after Usp26 deletion in vitro. BMSCs isolated from Usp26 cKO mice and their WT littermate controls were cultured in osteogenic, adipogenic, or chondrogenic media for different particular days. RT‐qPCR results demonstrated that osteogenic induction upregulated osteogenesis‐related genes, including osteocalcin (Ocn), alkaline phosphatase (Alp), and runt‐related transcription factor 2 (Runx2) (Figure 2H). Adipogenic induction upregulated adipogenesis‐related genes, such as CCAAT enhancer binding protein β (C/ebpβ), fatty acid binding protein 4 (Fabp4), and peroxisome proliferator‐activated receptor gamma (Pparg) (Figure 2K). Chondrogenic induction upregulated chondrogenesis‐related genes, including Aggrecan, Col2a1, and Sox9 in a time‐dependent manner in BMSCs from WT mice (Figure 2M). However, the expression of these genes was significantly reduced in the absence of Usp26. Furthermore, ALP staining and alizarin red S (ARS) staining results indicated a marked reduction in ALP activity and extracellular matrix mineralization in Usp26 cKO BMSCs (Figure 2F,G). Oil red O staining revealed a significant decrease in lipid droplet formation in BMSCs lacking USP26 (Figure 2I,J) while Alcian blue staining showed a decrease in chondrogenic differentiation in BMSCs with USP26 deletion (Figure 2L).

We further investigated the regulatory role of USP26 on the trilineage differentiation of BMSCs in vivo. Micro‐CT analysis revealed a significant decrease in bone mass in Usp26 cKO mice compared to the control mice, with bone parameters such as bone volume/tissue volume (BV/TV), trabecular number (Tb.N), trabecular thickness (Tb.Th), and bone mineral density (BMD) showing a significant decrease, while trabecular separation (Tb.Sp) showed an increase (Figure 2N,O). Cortical bone parameters, including BMD and cortical thickness (Ct.Th), also exhibited significant decreases (Figure S2A,B, Supporting Information). This indicates that osteogenic differentiation is significantly inhibited in Usp26 cKO mice. H&E staining of the femur showed a notable decrease in the number and area of adipocytes in Usp26 cKO mice (Figure 2P,Q). Moreover, IHC analysis of the adipose tissue marker FABP4 also demonstrated a significant decrease in Usp26 cKO mice (Figure 2R). The abnormal differentiation of BMSCs into chondrocytes or osteoblasts disrupts early skeletal development and leads to an osteopenic phenotype. Skeleton Alizarin red and Alcian blue co‐staining of the whole‐mount skeletons at E16.5 revealed that Usp26 cKO mice exhibited significant dwarfism during the embryonic period, and Alizarin red staining was less dense in Usp26 cKO mice, indicating impaired bone formation and development after Usp26 deletion (Figure 2S). Furthermore, a closer examination of femur regions with H&E staining and Alcian blue staining demonstrated that Usp26 cKO dampened the chondrogenesis of BMSCs, resulting in a significant decrease in chondrocyte density in the femurs (Figure 2T,U; Figure S2C,D, Supporting Information). Altogether, these data above indicate that a decrease in USP26 expression results in reduced proliferation ability and impaired multilineage differentiation potentials of BMSCs.

The multipotent differentiation properties of BMSCs show great potential for applications in tissue engineering and clinical treatments.^[^ ^3^, ^4^ ^]^ After observing that USP26 positively regulates the physiological formation of cartilage, we further evaluated the importance of USP26 in the regeneration of cartilage defects.

**Figure 3:**
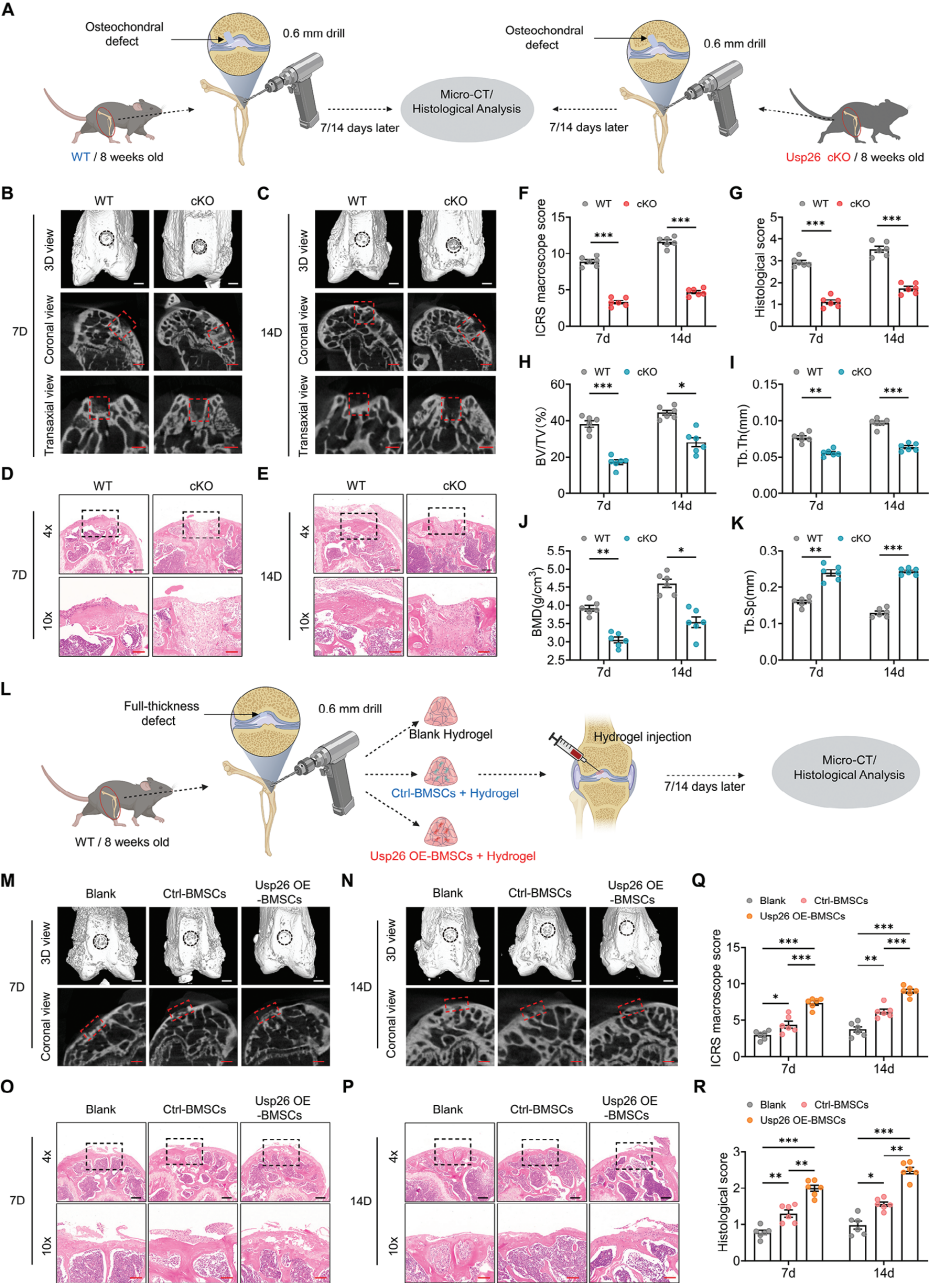
USP26 facilitated the repair of cartilage defects and regeneration. A) 0.6 mm holes were generated in the femoral bones of 8‐week‐old Usp26 cKO male mice and their WT littermate controls. The defects penetrated the bone marrow, and the defect bone samples were collected for Micro‐CT scanning and histological analysis after the surgery. B,C) Representative Micro‐CT images, coronal view, and transaxial view of the femur osteoarticular bone of 8‐week‐old Usp26 cKO male mice and their WT littermate controls at 7 and 14 days after surgery. n = 6 in each group. Scale bar, 500 µm (white) and 300 µm (red). D,E) Representative H&E staining images of the femoral bones of 8‐week‐old Usp26 cKO male mice and their WT littermate controls. n = 6 in each group. Scale bar, 200 µm (black) and 100 µm (red). F) Analysis of ICRS macroscope score for the harvested samples. n = 6 in each group. G) Histological score for the harvested samples. n = 6 in each group. H‐K) Quantitative analysis of the trabecular bone from Usp26 cKO male mice and their WT littermate controls, including BV/TV, Tb.Th, Tb.Sp, and BMD. n = 6 in each group. L) 0.6 mm holes were generated in femoral bones of 8‐week‐old male WT mice. The defects do not penetrate the subchondral bone, and the defects were filled with blank hydrogel, control BMSCs + hydrogel or Usp26 OE BMSCs + hydrogel. The defect bone samples were collected for Micro‐CT scanning and histological analysis after the surgery. M,N) Representative Micro‐CT images and coronal view of the femoral osteoarticular bone from different groups at 7 and 14 days after surgery. n = 6 in each group. Scale bar, 500 µm (white) and 300 µm (red). O‐P) Representative H&E staining images of femoral osteoarticular from different groups. n = 6 in each group. Scale bar, 200 µm (black) and 100 µm (red). Q) Analysis of ICRS macroscope score for the harvested samples. n = 6 in each group. R) Histological score for the harvested samples. n = 6 in each group. Mice age in A) to R), 8‐week‐old. BMSCs from mice at P5 were used in A) and L). Data are represented as mean ± SD. Statistical significance was determined by one‐way ANOVA. *p < 0.05, **p < 0.01, ***p < 0.001.

From the perspective of precision medicine, different types of cartilage defects require different repair methods.^[^ ^15^ ^]^ BMSCs are recruited to the defect area to partially enhance cartilage formation in cases of bone cartilage defects penetrating the bone marrow. In our study, we drilled holes in the femoral condyles of WT mice and Usp26 cKO mice in order to recruit BMSCs from the bone marrow to the defect area (**Figure** **3**A). Micro‐CT and histological analysis consistently showed that bone cartilage defects in WT mice began to heal at day 7 and were almost completely healed by day 14. However, the defects in Usp26 cKO mice were only partially healed by day 14 (Figure 3B–E). According to the International Cartilage Repair Society (ICRS) guidelines,^[^ ^16^ ^]^ the scores of Usp26 cKO mice at 7 and 14 days were significantly lower than those of WT mice, as confirmed by the histological scores of the harvested samples (Figure 3F,G). Analysis of BV/TV, Tb.Th, BMD, and Tb.Sp at the defect site showed that BMSCs from WT mice significantly promoted subchondral bone formation compared to those from Usp26 cKO mice (Figure 3H–K).

For cartilage defects that did not penetrate the tidal line, we injected blank hydrogel, hydrogel containing control BMSCs, and hydrogel containing BMSCs overexpressed Usp26 into the cartilage defects (Figure 3L). After 7 and 14 days, Micro‐CT and histological analysis, including H&E staining and Safranin O/Fast green staining, consistently showed that the BMSCs overexpressing Usp26 significantly promoted cartilage defect regeneration (Figure 3M–P; Figure S3A, Supporting Information), with the ICRS macroscope and histological scores further confirming the driving role of BMSCs with USP26 overexpression in cartilage regeneration (Figure 3Q,R).

BMSCs undergo functional decline and gradually lose their multipotency with age, which limits their therapeutic use.^[^ ^4a,c^ ^]^ To further investigate the function of Usp26 overexpression in senescent BMSCs for cartilage repair, blank hydrogel, hydrogel containing old BMSCs (O‐BMSCs) obtained from 20‐month‐old mice, and hydrogel containing O‐BMSCs with Usp26 overexpression were injected into full‐thickness cartilage defects to evaluate their effects on cartilage repair (Figure S4A, Supporting Information). The results of Micro‐CT and histological analysis, coupled with the ICRS macroscope and histological scores showed that Usp26 overexpression in O‐BMSCs significantly facilitated cartilage defects regeneration (Figure S4B–I, Supporting Information).

Collectively, these data demonstrated that USP26 not only regulated BMSCs differentiation to promote cartilage formation, but also played a role in regulating BMSC differentiation to promote cartilage regeneration.

### 2.3 USP26 Reverses Aging and Age‐Related Self‐Renewal and Multipotent Differentiation Declines of BMSCs

**Figure 4:**
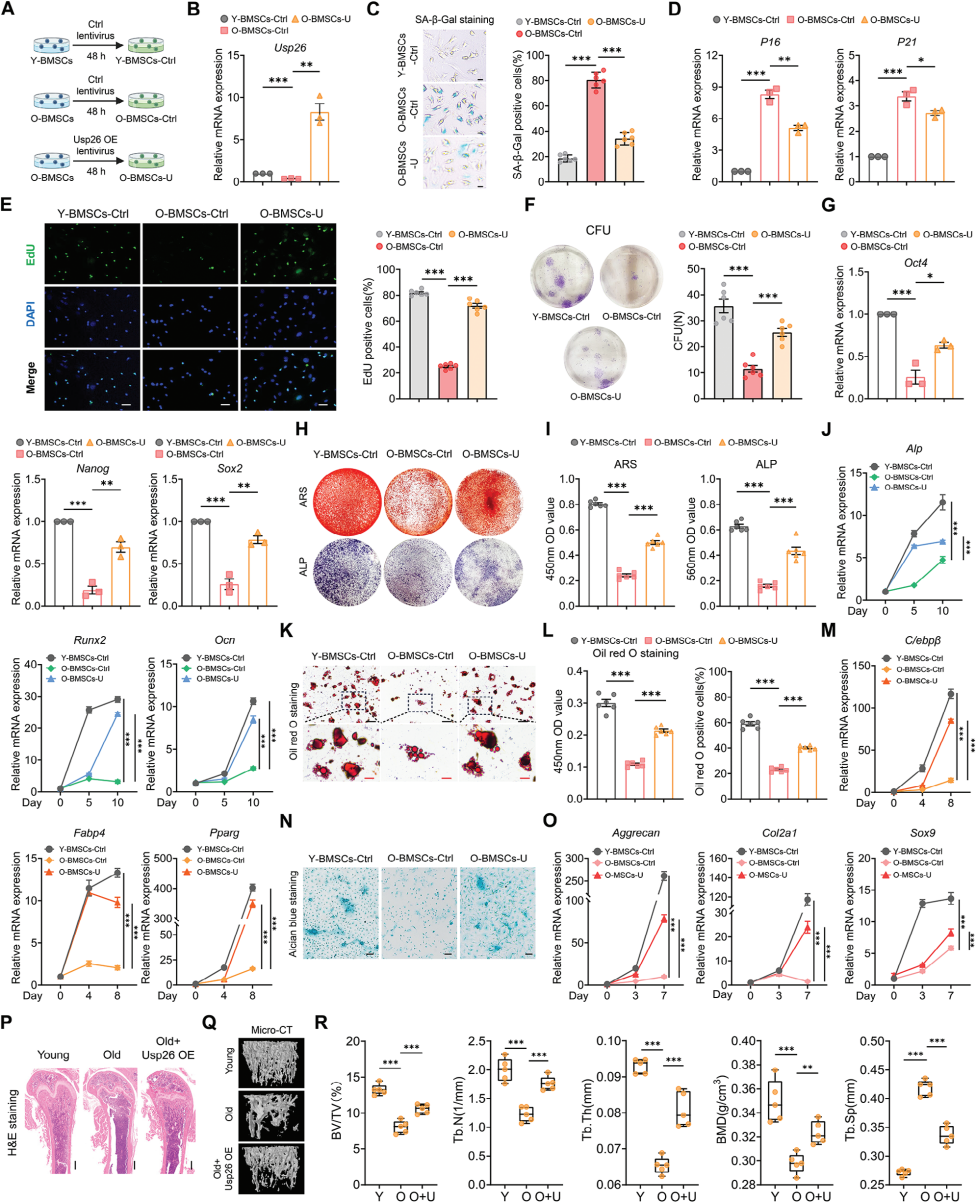
Supplementation of Usp26 reverses aging and age‐related self‐renewal and multipotent differentiation declines of BMSCs. A) Schematic diagram illustrating Y‐BMSCs and O‐BMSCs transfected with different lentivirus. B) qPCR analysis of Usp26 mRNA expressions in young BMSCs, O‐BMSCs, and O‐BMSCs after being treated with Usp26 overexpression lentivirus. n = 3 in each group. C) Representative SA‐β‐Gal staining images and percentage of SA‐β‐Gal positive cells from young BMSCs, O‐BMSCs, and O‐BMSCs after treated with Usp26 overexpression lentivirus. n = 6 in each group. Scale bar, 10 µm. D) qPCR analysis of P16 and P21 mRNA expressions in young BMSCs, O‐BMSCs, and O‐BMSCs after being treated with Usp26 overexpression lentivirus. n = 3 in each group. E) Representative EdU staining images and percentage of EdU positive cells from young BMSCs, O‐BMSCs, and O‐BMSCs after being treated with Usp26 overexpression lentivirus. n = 6 in each group. Scale bar, 20 µm. F) Representative images of CFU assay in young BMSCs, O‐BMSCs, and O‐BMSCs after being treated with Usp26 overexpression lentivirus. Colony number serves as a quantitative measurement. n = 6 in each group. G) qPCR analysis of Oct4, Nanog, and Sox2 mRNA expressions in young BMSCs, O‐BMSCs, and O‐BMSCs after being treated with Usp26 overexpression lentivirus. n = 3 in each group. H) Representative images of ARS and ALP from particular groups after 14 days of osteogenic induction. n = 6 in each group. I) Statistical analysis of the absorbance at 450 nm of ARS and the absorbance at 560 nm of ALP staining. n = 6 in each group. J) qPCR analysis of Alp, Runx2, and Ocn mRNA expressions in BMSCs from particular groups after different days (0, 5, and 10 days) of osteogenic induction. n = 3 in each group. K) Representative images of Oil red O staining from particular groups after 21 days of adipogenic differentiation. n = 6 in each group. Scale bar, 20 µm (black) and 5 µm (red). L) Statistical analysis of the absorbance at 450 nm of Oil red O staining and percentage of Oil red O positive cells from particular groups. n = 6 in each group. M) qPCR analysis of C/ebpβ, Fabp4, and Pparg mRNA expressions in BMSCs from particular groups after different days (0, 4, and 8 days) of adipogenic differentiation. n = 3 in each group. N) Representative images of Alcian blue staining of BMSCs from particular groups after 7 days of chondrogenic differentiation. n = 6 in each group. Scale bar, 25 µm. O) qPCR analysis of Aggrecan, Col2a1, and Sox9 mRNA expressions in BMSCs from particular groups after different days (0, 3, and 7 days) of chondrogenic differentiation. n = 3 in each group. P) Representative H&E staining images of the femurs from young mice (Y), old mice (O), and old mice treated with Usp26 overexpression adenovirus (O+U). n = 5 in each group. Scale bar, 500 µm. Q) Representative Micro‐CT images of the femurs from young mice, old mice, and old mice treated with Usp26 overexpression adenovirus. n = 5 in each group. R) Quantitative analysis of the trabecular bone from young mice, old mice, and old mice treated with Usp26 overexpression adenovirus, including BV/TV, Tb.N, Tb.Th, Tb.Sp, and BMD. n = 5 in each group. Young mice age, 2‐month‐old. Old mice age, 20‐month‐old. BMSCs from mice at P5 were used in B)‐O). Data are represented as mean ± SD. Statistical significance was determined by one‐way ANOVA in B), C), D), E), F), G), I), L), and R), or two‐way ANOVA in J), M), and O). *p < 0.05, **p < 0.01, ***p < 0.001.

After observing that decreased USP26 expression was associated with the aging of BMSCs and that USP26 deficiency resulted in decreased self‐renewal ability and impaired multilineage differentiation potentials of BMSCs, we next sought to investigate whether the Usp26 supplementation could reverse the aging and age‐related self‐renewal and multilineage differentiation declines of BMSCs. For this purpose, BMSCs collected from 2‐month‐old mice were used as young BMSCs (Y‐BMSCs), while BMSCs collected from 20‐month‐old mice were used as O‐BMSCs. The supplementation of Usp26 in O‐BMSCs was achieved through transfection with lentivirus carrying Usp26. Specifically, young BMSCs transfected with control lentivirus (Y‐BMSCs‐Ctrl), O‐BMSCs transfected with control lentivirus (O‐BMSCs‐Ctrl), and O‐BMSCs transfected with Usp26 overexpression lentivirus (O‐BMSCs‐U) (**Figure** **4**A).

In comparison to Y‐BMSCs, O‐BMSCs showed an increased number of β‐galactosidase‐positive cells and a significant elevation in the expression of aging markers P16 and P21 mRNA. However, when infected with lentivirus carrying Usp26, the expression of Usp26 mRNA in O‐BMSCs significantly increased (Figure 4B). Concurrently, the aging index of O‐BMSCs, including β‐galactosidase‐positive cells, P16, and P21 mRNA expression, significantly decreased (Figure 4C,D). Additionally, the results of EdU staining and CFU revealed significant recovery in the proliferative capacity of O‐BMSCs (Figure 4E,F).

In addition, the effect of Usp26 supplementation on the trilineage differentiation ability of O‐BMSCs was evaluated. Compared with Y‐BMSCs, O‐BMSCs exhibited decreased mRNA expression of the multipotency‐related transcription factors Nanog, Oct4, and Sox2. However, after infecting O‐BMSCs with lentivirus overexpressing Usp26, the mRNA expression of Nanog, Oct4, and Sox2 was significantly restored (Figure 4G). Moreover, the trilineage differentiation ability further indicated that the supplementation of Usp26 significantly enhanced the osteogenic, adipogenic, and chondrogenic differentiation abilities of O‐BMSCs, as evidenced by the upregulation of specific markers associated with each lineage (Figure 4H–O). Additionally, injection of Usp26 overexpression adenovirus into the bone marrow cavity significantly increased the bone mass in aged mice (Figure 4P–R).

To further confirm the ability of USP26 to restore aging‐related declines in self‐renewal, the self‐renewal of BMSCs over multiple passages was evaluated (Figure S5A, Supporting Information). Flow cytometry analysis of CD44^+^ and CD105^+^ cells in passages 1, 3, 5, and 7 showed that Y‐BMSCs‐Ctrl maintained a stable percentage of CD44^+^ and CD105^+^ cells while O‐BMSCs‐Ctrl exhibited a decline with increasing passages. In contrast, O‐BMSCs‐U showed resistance to this decline, indicating that Usp26 overexpression significantly promotes the self‐renewal ability of O‐BMSCs (Figure S5B, Supporting Information).

Furthermore, to determine whether Usp26 could restore the declines in differentiation potential of aged BMSCs over multiple passages, P7 Y‐BMSCs‐Ctrl, O‐BMSCs‐Ctrl, and O‐BMSCs‐U were induced for osteoblastic, adipogenic, and chondrogenic differentiation, respectively. The results demonstrated that Usp26 overexpression rescued the impaired trilineage differentiation ability of BMSCs at passage 7 (Figure S5C–J, Supporting Information). Moreover, the decreased expression of multipotency‐related genes such as Nanog, Oct4, and Sox2 in P7 O‐BMSCs was significantly restored (Figure S5K, Supporting Information).

Collectively, these data demonstrated that Usp26 supplementation reversed aging, age‐related self‐renewal, and multipotent differentiation declines of BMSCs.

### 2.4 USP26 Reverses Aging and Age‐Related Self‐Renewal and Multipotent Differentiation Declines of BMSCs by Improving Mitochondrial Homeostasis

**Figure 5:**
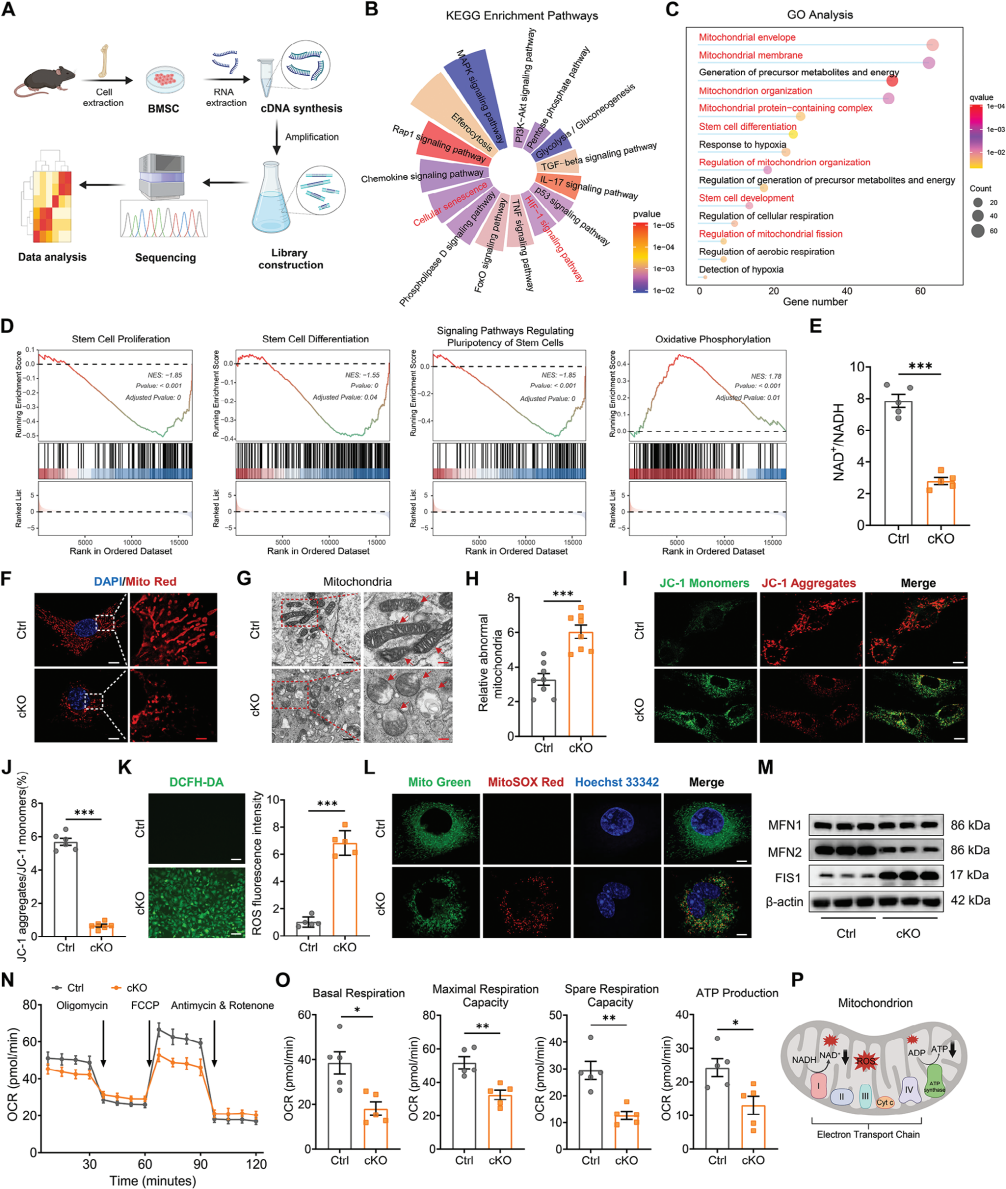
The decrease of USP26 resulted in impaired mitochondrial function of BMSCs. A) Schematic diagram depicting RNA‐Seq and downstream analysis. B) The rose chart of KEGG enrichment analysis for differentially expressed mRNA between BMSCs from control and cKO mice. C) The bubble chart of GO analysis for differentially expressed mRNA between BMSCs from control and cKO mice. D) GSEA showing significant differential enrichment of genes in pathways related to stem cell proliferation, stem cell differentiation, signaling pathways regulating the multipotency of stem cells, and oxidative phosphorylation. E) The ratio of NAD^+^/NADH in BMSCs from control and cKO mice. n = 5 in each group. F) Representative images of MitoTracker Red staining in BMSCs from control and cKO mice. n = 6 in each group. Scale bar, 2 µm (white) and 0.5 µm (red). G) Representative TEM images of mitochondria in BMSCs from control and cKO mice. n = 8 in each group. Scale bar, 500 nm (black) and 200 nm (red). H) Relative abnormal mitochondria rates in BMSCs from control and cKO mice. n = 8 in each group. I) Detection of JC‐1 monomers (green) and aggregates (red) by confocal fluorescence microscopy in BMSCs from control and cKO mice. n = 6 in each group. Scale bar, 4 µm. J) The ratio of JC‐1 aggregates/JC‐1 monomers in BMSCs from control and cKO mice. n = 6 in each group. K) Representative images of the DCFH‐DA assay showing intracellular ROS levels in BMSCs from control and cKO mice. ROS fluorescence intensity serves as a quantitative measurement. n = 5 in each group. Scale bar, 20 µm. L) Representative images of mtROS in BMSCs from control and cKO mice visualized with MitoSOX (red) staining. n = 5 in each group. Scale bar, 2 µm. M) Western blot analysis of MFN1, MFN2, and FIS1 protein levels in BMSCs from control and cKO mice. n = 3 in each group. N) Detection of the oxygen consumption rates (OCR) in BMSCs from control and cKO mice in response to indicated mitochondrial modulators (Oligomycin, FCCP, Antimycin & Rotenone). n = 5 in each group. O) Calculation of basal respiration, maximal respiration capacity, spare respiration capacity, and ATP production of BMSCs from control and cKO mice by the OCR values. n = 5 in each group. P) Schematic diagram depicting the impaired mitochondria. Mice age in A), E), F), G), H), I), J), K), L), M), N), and O), 2‐month‐old. BMSCs from mice at P5 were used in E)‐O). Data are represented as mean ± SD. Statistical significance was determined by two‐sided student's t test. *p < 0.05, **p < 0.01, ***p < 0.001.

Next, we delved into the molecular mechanisms underlying BMSC aging and age‐related self‐renewal and multipotent differentiation declines owing to decreased USP26 expression. RNA‐sequencing was conducted on BMSCs from Usp26 cKO mice and their littermate controls (**Figure** **5**A). Volcano plots illustrated 1050 upregulated genes and 738 downregulated genes in BMSCs from Usp26 cKO mice compared to the controls (Figure S6A,B and File 1, Supporting Information). Kyoto Encyclopedia of Genes and Genomes (KEGG) enrichment analysis showed notable variances in the cellular senescence signaling pathway in BMSCs following Usp26 deletion (Figure 5B). Gene Ontology (GO) analysis pinpointed significant differences in genes related to the mitochondrial envelope, mitochondrial membrane, mitochondrion organization, mitochondrial protein‐containing complex, and regulation of mitochondrial fission (Figure 5C). Additionally, gene set enrichment analysis (GSEA) of differentially expressed genes identified significant variations in pathways such as stem cell proliferation, stem cell differentiation, multipotency of stem cells, and oxidative phosphorylation (Figure 5D; Figure S6C, Supporting Information).

Several studies have highlighted the tight correlation between mitochondrial dysfunction and stem cell aging,^[^ ^17^ ^]^ indicating that Usp26 deletion could accelerate BMSCs aging by impacting mitochondrial homeostasis. Mitochondrial respiration and intracellular nicotinamide adenine dinucleotide (NAD)^+^ levels play a crucial role in cellular oxidative phosphorylation (OXPHOS).^[^ ^18^ ^]^ In comparison to the control, BMSCs from Usp26 cKO mice displayed a significant decrease in NAD^+^/NADH content (Figure 5E). MitoTracker Red staining exhibited heightened mitochondrial fragmentation (Figure 5F). Transmission electron microscopy (TEM) unveiled mitochondrial swelling, reduced or absent cristae, and a substantial increase in the number of abnormal mitochondria (Figure 5G,H). JC‐1 staining demonstrated Usp26 deletion disrupted mitochondrial membrane potential, with an uptick in green fluorescence and a decline in red fluorescence (Figure 5I,J). Furthermore, Usp26 deletion prompted a significant increase in reactive oxygen species (ROS) production in BMSCs (Figure 5K), depicted by elevated MitoSOX staining and oxidative stress damage to the mitochondria (Figure 5L). The expression of the fusion protein MFN2 was markedly decreased, whereas that of the fission protein FIS1 was elevated in BMSCs with Usp26 deletion (Figure 5M). Additionally, Usp26 deletion disrupted mitochondrial respiratory function, leading to lowered basal respiration, decreased maximal respiration capacity, reduced spare respiratory capacity, and a decline in ATP production (Figure 5N,O). In conclusion, these findings suggested that Usp26 deletion significantly compromised mitochondrial morphology and function (Figure 5P).

**Figure 6:**
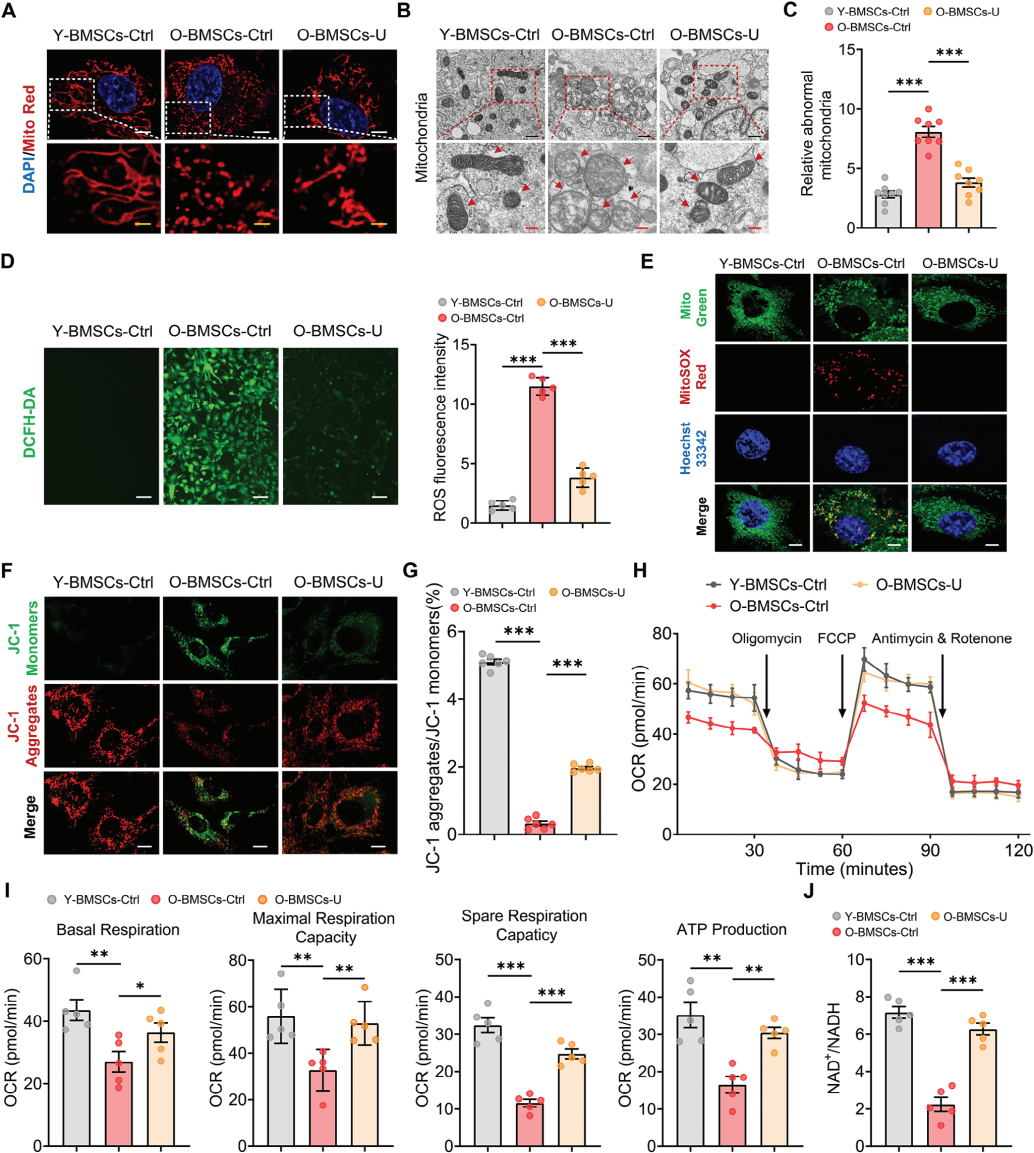
USP26 supplementation reversed age‐related declines in mitochondrial function of BMSCs. A) Representative images of mitochondria in young BMSCs, O‐BMSCs, and O‐BMSCs treated with Usp26 overexpression lentivirus visualized with MitoTracker Red staining. n = 5 in each group. Scale bar, 2 µm (white) and 0.5 µm (yellow). B) Representative TEM images of mitochondria in Young BMSCs, O‐BMSCs, and O‐BMSCs treated with Usp26 overexpression lentivirus. n = 8 in each group. Scale bar, 500 nm (black) and 200 nm (red). C) Relative abnormal mitochondria rates in BMSCs from particular groups. n = 8 in each group. D) Representative images of DCFH‐DA assay showing intracellular ROS levels in young BMSCs, O‐BMSCs, and O‐BMSCs treated with Usp26 overexpression lentivirus. ROS fluorescence intensity serves as a quantitative measurement. n = 5 in each group. Scale bar, 20 µm. E) Representative images of mtROS in young BMSCs, O‐BMSCs, and O‐BMSCs treated with Usp26 overexpression lentivirus visualized with MitoSOX (red) staining. n = 5 in each group. Scale bar, 2 µm. F) Detection of JC‐1 monomers (green) and aggregates (red) by confocal fluorescence microscopy in young BMSCs, O‐BMSCs, and O‐BMSCs treated with Usp26 overexpression lentivirus. n = 6 in each group. Scale bar, 4 µm. G) The ratio of JC‐1 aggregates/JC‐1 monomers in BMSCs from particular groups. n = 6 in each group. H) Detection of the OCR in young BMSCs, O‐BMSCs, and O‐BMSCs treated with Usp26 overexpression lentivirus in response to indicated mitochondrial modulators (Oligomycin, FCCP, Anitimycin & Rotenone). n = 5 in each group. I) Calculation of basal respiration, maximal respiration capacity, spare respiration capacity, and ATP production of BMSCs from particular groups by the OCR values. n = 5 in each group. J) The ratio of NAD^+^/NADH in young BMSCs, O‐BMSCs, and O‐BMSCs treated with Usp26 overexpression lentivirus. n = 5 in each group. Young mice age, 2‐month‐old. Old mice age, 20‐month‐old. BMSCs from mice at P5 were used in A)‐J). Data are represented as mean ± SD. Statistical significance was determined by one‐way ANOVA. *p < 0.05, **p < 0.01, ***p < 0.001.

To further investigate whether decreased expression of USP26 was responsible for the impaired mitochondrial function in aged BMSCs, we aimed to restore USP26 expression and determine its effects on mitochondrial function. The results presented in **Figure** **6** demonstrate that reinstating USP26 expression significantly improved mitochondrial morphology integrity (Figure 6A), reduced the number of abnormal mitochondria in aged BMSCs (Figure 6B,C), lowered intracellular ROS levels (Figure 6D), decreased mitochondrial oxidative damage (Figure 6E), and restored mitochondrial membrane potential (Figure 6F,G). Additionally, reintroducing USP26 expression in aged BMSCs enhanced mitochondrial respiratory function, increased intracellular ATP production (Figure 6H,I), and raised NAD^+^/NADH levels (Figure 6J). Collectively, these data demonstrated that reintroducing Usp26 expression in aging BMSCs significantly improved mitochondrial morphology and respiratory function.

### 2.5 USP26 Regulates the Mitochondrial Function of BMSCs through SIRT2

**Figure 7:**
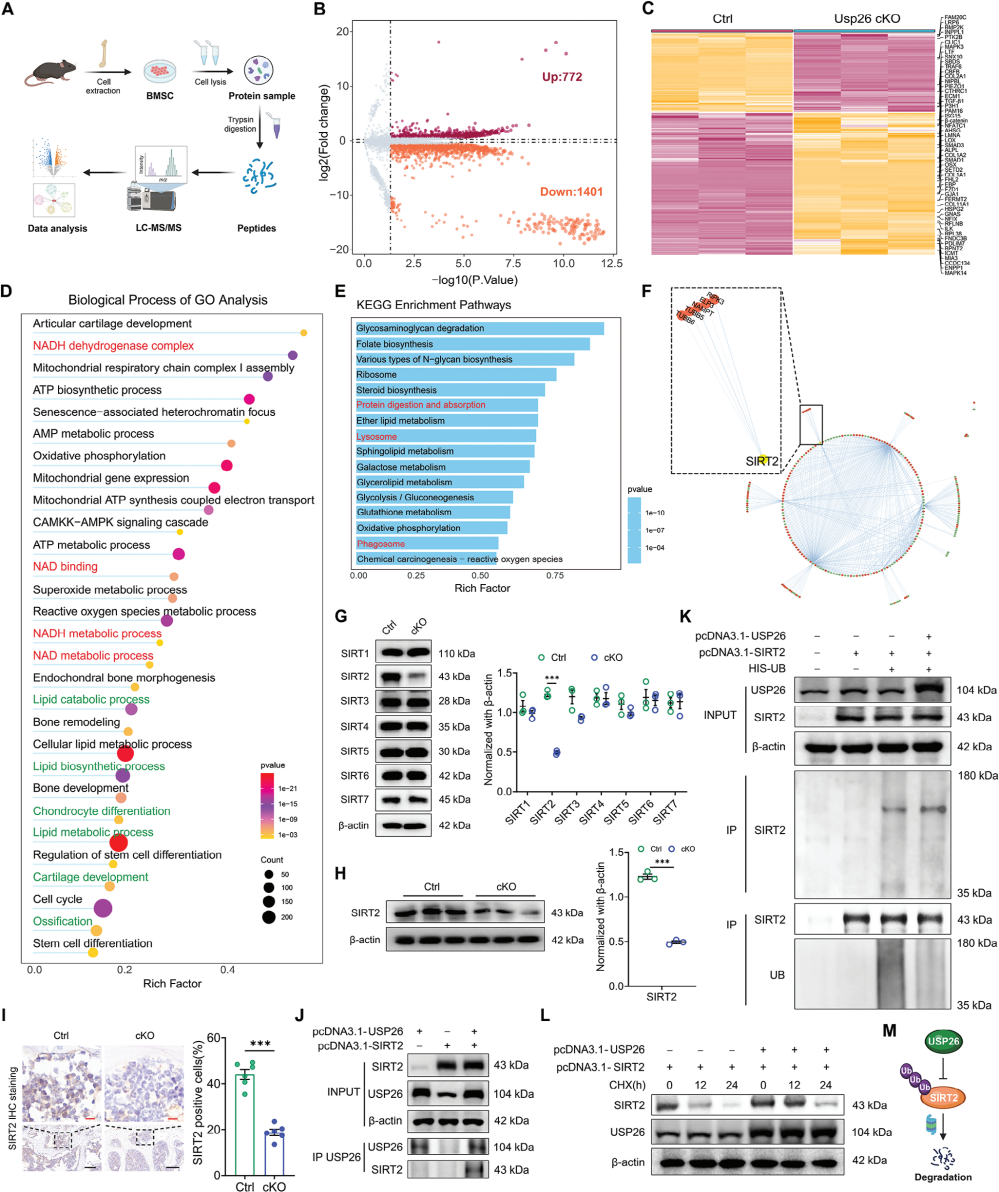
The decrease of USP26 results in the ubiquitination degradation of SIRT2 in BMSCs. A) Schematic diagram depicting proteomics and downstream analysis. B) Volcano plots showing differentially expressed proteins in BMSCs from controls and cKO mice. The purple and orange dots represent the up‐regulated and down‐regulated proteins, respectively. C) Heatmap showing differentially expressed proteins of BMSCs from controls and cKO mice. D) The bubble chart of GO analysis for differentially expressed biological process of BMSCs from control and cKO mice. E) Bar plots showing KEGG enrichment analysis for differentially expressed proteins of BMSCs from controls and cKO mice. F) A network diagram of interactions among differentially expressed proteins in BMSCs from control and cKO mice. G) Western‐blot analysis and quantification of SIRT1, SIRT2, SIRT3, SIRT4, SIRT5, SIRT6, and SIRT7 protein levels in BMSCs from controls and cKO mice. n = 3 in each group. H) Western‐blot analysis and quantification of SIRT2 protein level in bone tissue from femurs of control and cKO mice. n = 3 in each group. I) Representative IHC staining of SIRT2 of the femurs from control and cKO mice. n = 6 in each group. Scale bar, 100 µm (black) and 10 µm (red). J) Co‐immunoprecipitation of USP26 with ectopically expressed SIRT2 in HEK‐293 T cells. K) Overexpression of Usp26 decreases the level of ubiquitinated of SIRT2 and increases the expression of SIRT2 protein. L) Western‐blot analysis of the protein level of SIRT2 in HEK‐293 T cells with or without Usp26 overexpression and treated with cycloheximide (CHX) for indicated time intervals. M) The schematic graph reflects the underlying mechanisms of USP26 in decreasing SIRT2 protein degradation by reducing the level of ubiquitinated SIRT2. Mice age in A), G), H), and I), 2‐month‐old. BMSCs from mice at P5 were used in G) and H). Data are represented as mean ± SD. Statistical significance was determined by two‐sided student's t test. ***p < 0.001.

As a deubiquitylating enzyme, USP26 plays a biological role in stabilizing various targets.^[^ ^9^ ^]^ We next sought to investigate the potential substrates involved in USP26‐mediated mitochondrial homeostasis in BMSCs. Liquid chromatography‐tandem mass spectrometry (LC‐MS/MS)‐based proteomics was utilized to compare the proteomes of control and Usp26 cKO BMSCs (**Figure** **7**A). Volcano plots of the differentially expressed proteins displayed a total of 772 upregulated proteins and 1401 downregulated proteins (Figure 7B; File 2, Supporting Information), including lipid receptor LRP6, chondrocyte marker COL2A1, osteoblast marker COL1A1, and OSX (Figure 7C). KEGG pathway analysis and GSEA identified significant differences in cellular protein degradation pathways post Usp26 deletion (Figure 7E; Figure S7B, Supporting Information). GO analysis of differentially expressed biological processes revealed significant variances in processes such as lipid metabolic process, chondrocyte differentiation, cartilage development, and ossification (Figure 7D). Reactome pathway analysis also indicated significant differences in the mitochondria‐related signaling pathways, including the TCA cycle, mitotic cell cycle, and respiratory electron transport. (Figure S7A, Supporting Information). These findings further support the notion that Usp26 deletion in BMSCs results in mitochondrial dysfunction and impairs the multilineage differentiation of BMSCs.

The network diagram displaying the interactions among the differentially expressed proteins revealed the top nine proteins with the most interactions: NF‐KB1, PRKACA, PRKACB, TRP53BP1, SIRT2, PRKAA1, PRKAB1, PRKAR1A, and PRKAR2A (Figure 7F). NF‐KB1, a transcription factor, primarily regulates immune responses^[^ ^19^ ^]^; TRP53BP1, also known as tumor protein P53 binding protein 1, participates in DNA damage response and cell cycle regulation^[^ ^20^ ^]^; PRKACA and PRKACB are catalytic subunits of cAMP‐dependent protein kinase (PKA)^[^ ^21^ ^]^; PRKAA1 and PRKAB1, also known as AMPK alpha 1and AMPK beta 1 respectively, are subunits of the AMP‐activated protein kinase (AMPK)^[^ ^22^ ^]^; PRKAR1A and PRKAR2A are regulatory subunits of cAMP‐dependent protein kinase^[^ ^23^ ^]^; these proteins are involved in cAMP‐PKA signaling pathways regulation, with dysregulation linked to various diseases including cancer and metabolic disorders.^[^ ^24^ ^]^ SIRT2, a NAD‐dependent protein deacetylase belonging to the SIRT family,^[^ ^25^ ^]^ plays a vital role in maintaining mitochondrial homeostasis,^[^ ^26^ ^]^ lifespan extension, and aging inhibition,^[^ ^27^ ^]^ in addition to its role in the early lineage commitment of mouse embryonic stem cells. Combined with the GO analysis findings involving NAD^+^/NADH metabolic processes, NAD binding, and NADH dehydrogenase complex (Figure 7D), the observation of decreased NAD^+^/NADH content in BMSCs from Usp26 cKO mice (Figure 5E), lead us to hypothesize that USP26 regulates BMSC mitochondrial function, self‐renewal, and multipotent differentiation through the modulation of SIRT2 protein stability. This is in line with the significant differences in the NAD^+^/NADH metabolic processes, which govern the activity of sirtuins (SIRTs). Additionally, results of the western blot further demonstrated a notable decrease in the protein expression of SIRT2 in BMSCs and femoral tissues of Usp26 cKO mice (Figure 7G,H). Furthermore, immunohistochemistry of femoral tissues from both controls and Usp26 cKO mice also revealed a significant decrease in the number of SIRT2 positive cells in Usp26 cKO mice (Figure 7I). SIRTs, including SIRT1 through SIRT7, are NAD^+^‐dependent histone deacetylases that are involved in various biological processes.^[^ ^28^ ^]^ SIRT2 is the only Sirtuin protein predominantly found in the cytoplasm; it is also present in the mitochondria and nucleus.^[^ ^25^ ^]^ In addition to rescuing mitochondrial integrity, SIRT2 supplementation enhances bioenergetics by deacetylating mitochondrial proteins to increase ATP generation in the axons.^[^ ^26^ ^]^ Therefore, we speculated that decreased SIRT2 expression may be responsible for the impaired mitochondrial function observed in Usp26 cKO BMSCs. Co‐immunoprecipitation (Co‐IP) assays revealed SIRT2 enrichment in complexes precipitated with antibodies against USP26 in HEK‐293 T cells (Figure 7J). Furthermore, the overexpression of USP26 significantly decreased the level of ubiquitinated SIRT2 (Figure 7K) and led to slower SIRT2 protein degradation in the presence of cycloheximide, a protein translation inhibitor (Figure 7L). These findings suggested that USP26 reduced SIRT2 protein degradation by decreasing the ubiquitination level of SIRT2 (Figure 7M).

**Figure 8:**
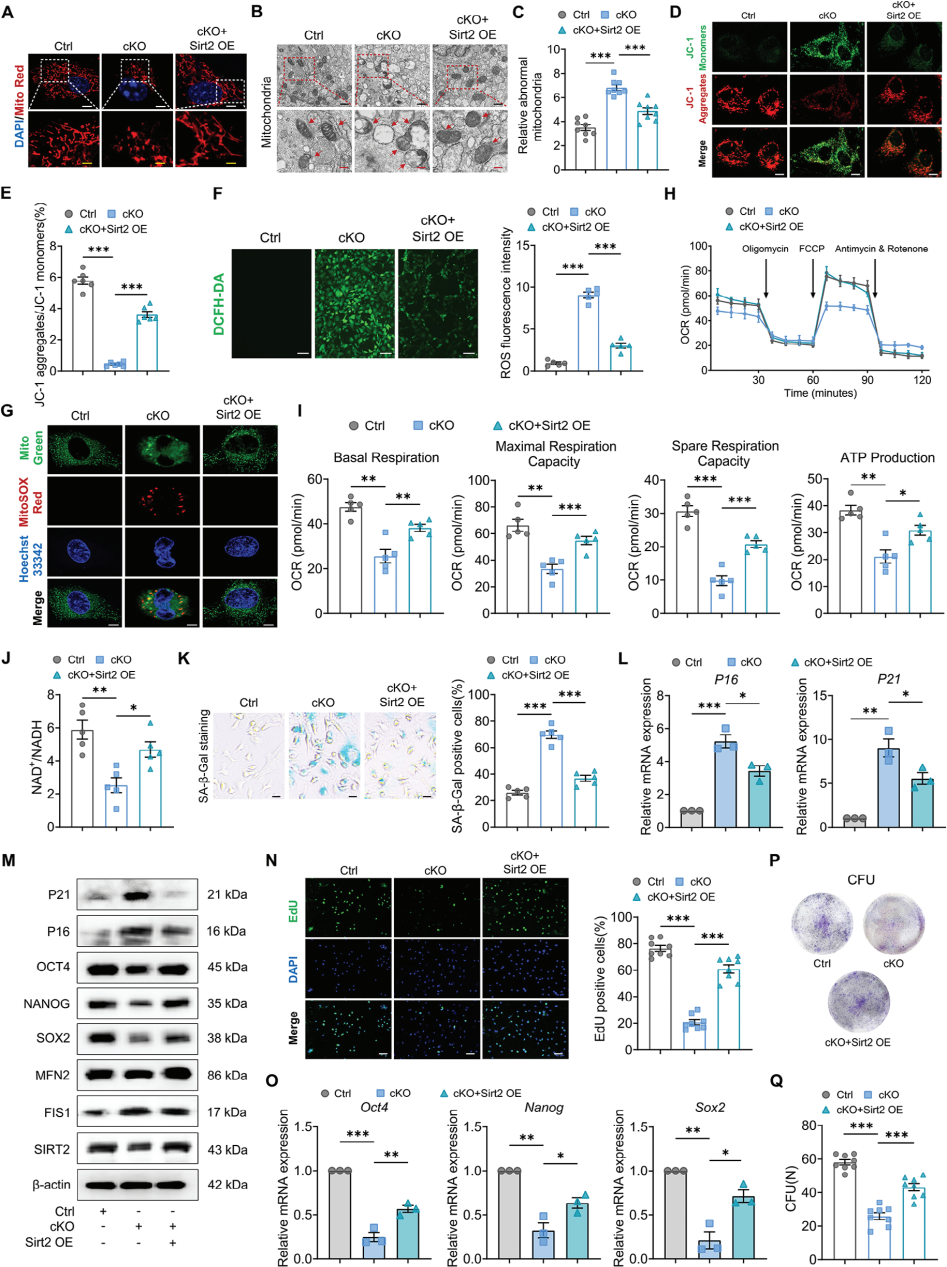
The decrease in the expression of SIRT2 was responsible for the impaired mitochondrial function and the decline in self‐renewal and multipotency of Usp26 cKO BMSCs. A) Representative images of mitochondria from control BMSCs, cKO BMSCs, and cKO BMSCs treated with SIRT2 overexpression lentivirus visualized with MitoTracker Red staining. n = 5 in each group. Scale bar, 2 µm (white) and 0.5 µm (yellow). B) Representative TEM images of mitochondria from control BMSCs, cKO BMSCs, and cKO BMSCs treated with SIRT2 overexpression lentivirus. n = 8 in each group. Scale bar, 500 nm (black) and 200 nm (red). C) Relative abnormal mitochondria rates in BMSCs from particular groups. n = 8 in each group. D) Detection of JC‐1 monomers (green) and aggregates (red) by confocal fluorescence microscopy in control BMSCs, cKO BMSCs, and cKO BMSCs treated with SIRT2 overexpression lentivirus. n = 6 in each group. Scale bar, 4 µm. E) The ratio of JC‐1 aggregates/JC‐1 monomers in BMSCs from particular groups. n = 6 in each group. F) Representative images of the DCFH‐DA assay showing intracellular ROS levels in BMSCs from particular groups. ROS fluorescence intensity serves as a quantitative measurement. n = 5 in each group. Scale bar, 20 µm. G) Representative images of mtROS in control BMSCs, cKO BMSCs, and cKO BMSCs treated with SIRT2 overexpression lentivirus visualized with MitoSOX (red) staining. n = 5 in each group. Scale bar, 2 µm. H) Detection of the OCR of control BMSCs, cKO BMSCs, and cKO BMSCs treated with SIRT2 overexpression lentivirus in response to indicated mitochondrial modulators (Oligomycin, FCCP, Antimycin & Rotenone). n = 5 in each group. I) Calculation of basal respiration, maximal respiration capacity, spare respiration capacity, and ATP production of BMSCs from particular groups by the OCR values. n = 5 in each group. J) The ratio of NAD^+^/NADH from control BMSCs, cKO BMSCs, and cKO BMSCs treated with Sirt2 overexpression lentivirus. n = 5 in each group. K) Representative SA‐β‐Gal staining images and percentage of SA‐β‐Gal positive cells of control BMSCs, cKO BMSCs and cKO BMSCs treated with SIRT2 overexpression lentivirus. n = 5 in each group. Scale bar, 10 µm. L) qPCR analysis of P16 and P21 mRNA expressions in BMSCs from particular groups. n = 3 in each group. M) Western‐blot analysis of P21, P16, OCT4, NANOG, SOX2, MFN2, FIS1, and SIRT2 protein levels in BMSCs from particular groups. n = 3 in each group. N) Representative EdU staining images and percentage of EdU positive cells of control BMSCs, cKO BMSCs, and cKO BMSCs treated with Sirt2 overexpression lentivirus. n = 8 in each group. Scale bar, 20 µm. O) qPCR analysis of Oct4, Nanog, and Sox2 mRNA expressions in BMSCs from particular groups. n = 3 in each group. P) Representative images of CFU in BMSCs from particular groups are shown. Q) Colony number serves as a quantitative measurement. n = 8 in each group. Mice age in A) to Q), 2‐month‐old. Data are represented as mean ± SD. BMSCs from mice at P5 were used in A)‐Q). Statistical significance was determined by one‐way ANOVA. *p < 0.05, **p < 0.01, ***p < 0.001.

To determine whether decreased SIRT2 expression was responsible for the impaired mitochondrial function observed in Usp26 cKO BMSCs, SIRT2 was reintroduced into Usp26 cKO BMSCs by transfecting them with lentivirus carrying Sirt2. As shown in **Figure** **8**A–E, the supplementation of Sirt2 significantly improved mitochondrial integrity (Figure 8A), greatly reduced the number of abnormal mitochondria (Figure 8B,C), and promoted the stability of mitochondrial membrane potential in Usp26 cKO BMSCs (Figure 8D,E). Following the deletion of USP26 in BMSCs, there was a significant increase in intracellular ROS levels, but the reintroduction of SIRT2 significantly decreased both intracellular ROS levels and mtROS levels (Figure 8F,G). Correspondingly, the respiratory function of the mitochondria in Usp26 cKO BMSCs significantly improved in response to SIRT2 reintroduction (Figure 8H,I). Additionally, the decrease in NAD^+^/NADH content caused by Usp26 cKO was significantly alleviated (Figure 8J). In line with the improvement in mitochondrial function, we found that SIRT2 overexpression significantly reduced cell aging in Usp26 cKO BMSCs and promoted cell proliferation and the expression of multipotency‐related genes (Figure 8K–Q).

To further confirm that USP26 regulates the aging, self‐renewal, multipotency, and mitochondrial function of BMSCs through SIRT2, lentiviruses containing Sirt2 shRNA (shSirt2) was introduced into the Usp26 cKO BMSCs with Usp26 overexpression (Usp26 OE). As shown in Figure S8A–G (Supporting Information), Usp26 overexpression significantly alleviated cell aging in Usp26 cKO BMSCs and promoted cell self‐renewal and the expression of multipotency‐related genes. However, these processes were significantly reversed by Sirt2 shRNA (Figure S8, Supporting Information). Moreover, Usp26 overexpression significantly improved mitochondrial integrity, reduced the number of abnormal mitochondria, promoted the stability of mitochondrial membrane potential, decreased both intracellular ROS levels and mtROS levels in Usp26 cKO BMSCs. Nevertheless, these processes were notably impaired by Sirt2 shRNA (Figure S9A–H, Supporting Information). In line with this, the improvement in respiratory function of mitochondria and the rescue in NAD^+^/NADH content observed in Usp26 cKO BMSCs induced by Usp26 overexpression were all significantly impaired by Sirt2 shRNA (Figure S9I–K, Supporting Information). Taken together, these data demonstrated that USP26 regulated the aging, self‐renewal, multipotency, and mitochondrial function of BMSCs through SIRT2.

### 2.6 Decreased USP26 Expression in Aged BMSCs Resulted from Reduced HIF‐1α Expression

**Figure 9:**
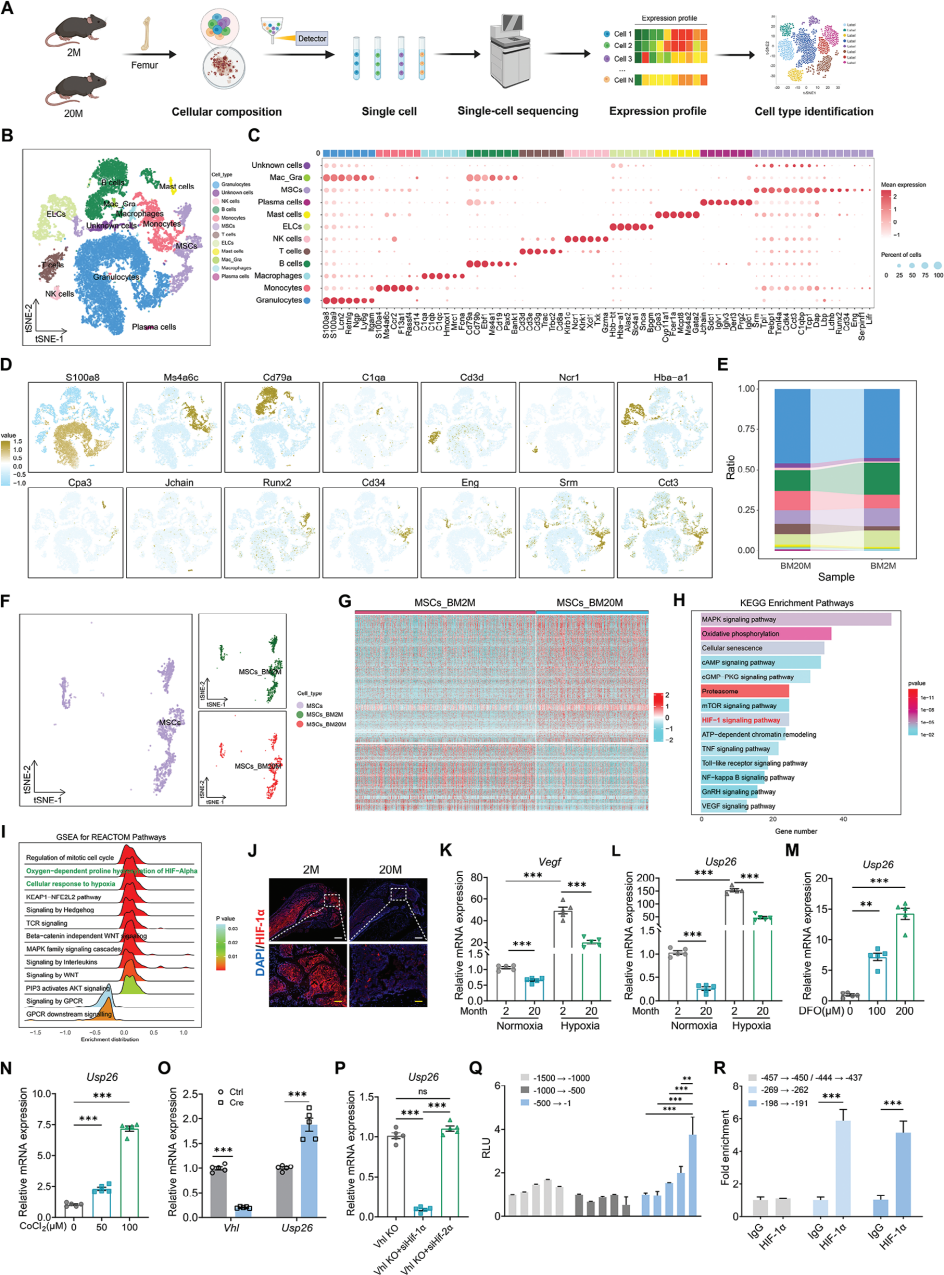
Decreased USP26 expression in aged BMSCs results from reduced HIF‐1α expression. A) Schematic diagram depicting Single‐cell RNA‐Seq and downstream analysis. B) Bone marrow cells of 2‐month‐old and 20‐month‐old mice were isolated for Single‐cell RNA‐seq analysis. t‐SNE plots showing all cell clusters identified using the computational pipeline. C) The bubble chart of the average expression of canonical marker genes for different cell types. D) t‐SNE plots showing the average expression of canonical marker genes for different cell types. E) The proportion of cell types in the single‐cell transcriptome data. F) t‐SNE plots showing BMSCs from 2‐month‐old and 20‐month‐old mice. G) Heatmap of differentially expressed mRNA in BMSCs from 2‐month‐old and 20‐month‐old mice. H) Bar plots showing KEGG enrichment analysis for differentially expressed mRNA in BMSCs from 2‐month‐old and 20‐month‐old mice. I) The peak chart of GSEA analysis for REACTOM Pathways. J) Representative immunofluorescent images of HIF‐1α from the femurs of 2‐month‐old and 20‐month‐old mice. n = 5 in each group. Scale bar, 500 µm (white) and 125 µm (yellow). K) qPCR analysis of Vegf mRNA expressions in BMSCs from 2‐month‐old and 20‐month‐old mice after cultured with normoxia or hypoxia conditions. n = 5 in each group. L) qPCR analysis of Usp26 mRNA expressions in BMSCs from 2‐month‐old and 20‐month‐old mice after cultured with normoxia or hypoxia conditions. n = 5 in each group. M) qPCR analysis of Usp26 mRNA expressions of BMSCs after being treated with different concentrations of DFO (0, 100, and 200 µM). n = 5 in each group. N) qPCR analysis of Usp26 mRNA expressions of BMSCs after treated with different concentrations of CoCl_2_ (0, 50, and 100 µM). n = 5 in each group. O) qPCR analysis of Vhl and Usp26 mRNA expressions in BMSCs following the treatment with control or Vhl Cre adenoviruses. n = 5 in each group. P) qPCR analysis of Usp26 mRNA expressions of BMSCs in different groups. n = 5 in each group. Q) Dual‐luciferase reporter assays showing the effects of different truncated mutants on Usp26 promoter transcriptional activity. n = 5 in each group. R) ChIP‐qPCR shows the binding sites in HIF‐1α protein and Usp26 promoter. n = 3 in each group. Young mice age, 2‐month‐old. Old mice age, 20‐month‐old. BMSCs from mice at P5 were used in K)‐P). Data are represented as mean ± SD. Statistical significance was determined by two‐way ANOVA in K) and L), one‐way ANOVA in M), N), P), and Q), and two‐sided student's t test in O) and R). **p < 0.01, ***p < 0.001.

Having observed a correlation between decreased USP26 expression and aging of BMSCs, as well as the promotion of cell aging through impaired mitochondrial function via SIRT2 in the absence of USP26, we delved further into the molecular mechanisms behind reduced USP26 expression in aged BMSCs. Our investigation involved Single‐cell RNA‐sequencing on bone marrow cells from mice aged 2 months (2 M) and 20 months (20 M) (**Figure** **9**A). The T‐distributed stochastic neighbor embedding (t‐SNE) plot illustrated a division of bone marrow cell types into 12 clusters, including Granulocytes, monocytes, macrophages, B cells, T cells, NK cells, ELCs, Mast cells, Plasma cells, MSCs, Mac_Gra, and other unidentified cells (Figure 9B). Different gene expression profiles were identified within these cell types, such as high expression of S100a8 in macrophages, and high expression of Cd34 and Srm in BMSCs (Figure 9C,D; Figure S10A and File 3, Supporting Information). The proportion of BMSCs in the total bone marrow cavity cells changed from 11% in the 2 M mice to 8% in the 20 M mice (Figure 9E). BMSCs were selected for further analysis, with the t‐SNE plot showcasing purple representing total BMSCs from 2 M and 20 M femurs, green representing 2 M BMSCs, and red representing 20 M BMSCs (Figure 9F). Differential gene analysis of these two clusters was conducted (Figure 9G; Figure S10D and File 4, Supporting Information), where GO analysis (Figure S10B, Supporting Information), KEGG enrichment pathway analysis (Figure 9H), REACTOM Pathways (Figure S10C, Supporting Information), and GSEA (Figure 9I) all displayed significant differences in genes related to the response to hypoxia and the HIF‐1α signaling pathway. Furthermore, GSEA of differentially expressed proteins between control and Usp26 cKO mice also showed significant variations in the pathway of Cellular response to hypoxia (Figure S7C, Supporting Information).

The low oxygen/HIF‐1α pathway is known to regulate the aging and differentiation processes of BMSCs.^[^ ^29^ ^]^ Evidence suggests that reduced or loss of HIF‐1α expression often results in aging, proliferation inhibition, and differentiation hindrance in BMSCs.^[^ ^30^ ^]^ It has been observed that elderly bone tissue exhibits down‐regulation of HIF‐1α and decreased responsiveness to low oxygen.^[^ ^31^ ^]^ Consistent with these findings, our study identified significant decreases in HIF‐1α expression in elderly bone tissues (Figure 9J) and decreased responsiveness to low oxygen in aged BMSCs isolated from mice at 20 M mice, as evidenced by reduced Vegf expression (Figure 9K). Additionally, the capacity of aged BMSCs to express Usp26 in response to low‐oxygen environment was notably diminished (Figure 9L). Therefore, these results suggest that the decreased HIF‐1α expression in aging BMSCs may contribute to the reduced expression of Usp26.

To investigate the regulatory role of HIF‐1α on USP26 expression in BMSCs, hypoxia‐mimicking compounds DFO^[^ ^32^ ^]^ and CoCl_2_^[^ ^33^ ^]^ were utilized. The results presented in Figure 9M,N indicated that DFO and CoCl_2_ can induce the expression of Usp26 in a dose‐dependent manner. Furthermore, the activation of the HIF‐1α pathway by Vhl deletion significantly increased Usp26 expression (Figure 9O). Vhl deletion prevents the degradation of HIF‐1α and HIF‐2α. It was observed that the knockout of Hif‐1α, rather than Hif‐2α, significantly inhibited the upregulation of Usp26 expression induced by Vhl knockout in BMSCs (Figure 9P).

As HIF‐1α accumulates and translocates into the nucleus, it forms a dimer with the HIF‐1β subunit via its bHLH‐PAS domain and binds to the promoter region of target genes, thereby enhancing their expression.^[^ ^34^ ^]^ Analysis of the mouse Usp26 promoter sequence using the JASPAR core database revealed the presence of five putative binding sites for Hif‐1α at −1 — ‐500 bp, 2 putative binding sites for Hif‐1α at −500 — ‐1000 bp, and five putative binding sites for Hif‐1α at −1000 — ‐1500 bp in the promoter region of Usp26. To further investigate which predicted binding site is essential for Usp26 promoter regulation by HIF‐1α, −1 — ‐500 bp, −500 — ‐1000 bp, and −1000 — ‐1500 bp promoter regions of the mouse USP26 were constructed. Dual‐luciferase reporter gene assays revealed that HIF‐1α mainly enhances the transcriptional activity of the −1 — ‐500 bp promoter region of Usp26 (Figure 9Q). Further ChIP‐qPCR results showed that HIF‐1α mainly binds to the −191— ‐198 bp and −262— ‐269 bp regions of the Usp26 promoter to enhance Usp26 transcriptional activity (Figure 9R). Collectively, these data indicated that the reduced expression of Usp26 in aged BMSCs resulted from decreased expression of HIF‐1α.

### 2.7 Activation of the HIF‐1α‐USP26 Pathway Combats Aging and Improves the Self‐Renewal and Multipotent Differentiation of Aged BMSCs

**Figure 10:**
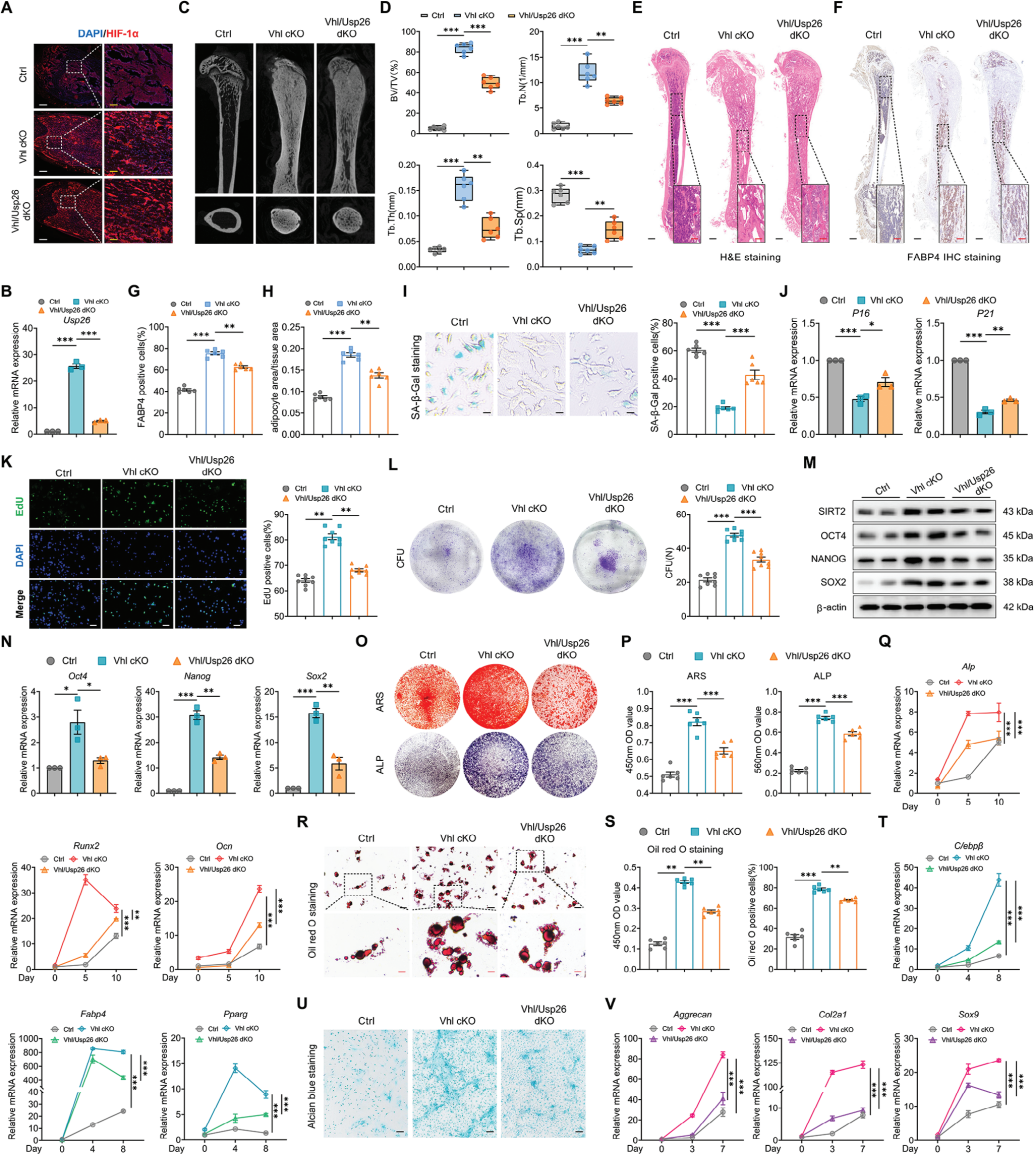
Activation of the HIF‐1α‐USP26 pathway combats aging and improves the self‐renewal and multipotent differentiation of aged BMSCs. A) Representative immunofluorescent images of HIF‐1α of the femurs from control, Vhl cKO, and Vhl/Usp26 dKO mice. n = 5 in each group. Scale bar, 500 µm (white) and 125 µm (yellow). B) qPCR analysis of Usp26 mRNA expression in BMSCs from control, Vhl cKO, and Vhl/Usp26 dKO mice. n = 3 in each group. C) Representative Micro‐CT images of the femurs from control, Vhl cKO, and Vhl/Usp26 dKO mice. n = 6 in each group. D) Quantitative analysis of the trabecular bone from control, Vhl cKO, and Vhl/Usp26 dKO mice, including BV/TV, Tb.N, Tb.Th, and Tb.Sp. n = 6 in each group. E) Representative H&E staining images of the femurs from control, Vhl cKO, and Vhl/Usp26 dKO mice. n = 6 in each group. Scale bar, 500 µm (black) and 100 µm (red). F) Representative IHC staining of FABP4 in the femurs from control, Vhl cKO, and Vhl/Usp26 dKO mice. n = 6 in each group. Scale bar, 500 µm (black) and 100 µm (red). G) FABP4 positive cells number as a quantitative measurement. n = 6 in each group. H)Area of adipocytes per tissue area measured based on H&E staining images. n = 6 each group. I) Representative SA‐β‐Gal staining images and percentage of SA‐β‐Gal positive cells in BMSCs from control, Vhl cKO, and Vhl/Usp26 dKO mice. n = 6 in each group. Scale bar, 10 µm. J) qPCR analysis of P16 and P21 mRNA expressions in BMSCs from control, Vhl cKO, and Vhl/Usp26 dKO mice. n = 3 in each group. K) Representative EdU staining images and percentage of EdU positive cells in BMSCs from control, Vhl cKO, and Vhl/Usp26 dKO mice. n = 8 in each group. Scale bar, 20 µm. L) Representative images of CFU in BMSCs from control, Vhl cKO, and Vhl/Usp26 dKO mice. Colony number serves as a quantitative measurement. n = 8 in each group. M) Western‐blot analysis of SIRT2, OCT4, NANOG, and SOX2 protein levels in BMSCs from control, Vhl cKO, and Vhl/Usp26 dKO mice. n = 3 in each group. N) qPCR analysis of Oct4, Nanog, and Sox2 mRNA expressions in BMSCs from control, Vhl cKO, and Vhl/Usp26 dKO mice. n = 3 in each group. O) Representative images of ARS and ALP of BMSCs from control, Vhl cKO, and Vhl/Usp26 dKO mice after 14 days of osteogenic differentiation. n = 6 in each group. P) Statistical analysis of the absorbance at 450 nm of ARS and the absorbance at 560 nm of ALP staining. n = 6 in each group. Q) qPCR analysis of Alp, Runx2, and Ocn mRNA expressions in BMSCs from control, Vhl cKO, and Vhl/Usp26 dKO mice after different days (0, 5, and 10 days) of osteogenic differentiation. n = 3 in each group. R) Representative images of Oil red O staining of BMSCs from control, Vhl cKO, and Vhl/Usp26 dKO mice after 21 days of adipogenic differentiation. n = 6 in each group. Scale bar, 20 µm (black) and 5 µm (red). S) Statistical analysis of the absorbance at 450 nm of Oil red O staining and percentage of Oil red O positive cells in BMSCs from control, Vhl cKO, and Vhl/Usp26 dKO mice. n = 6 in each group. T) qPCR analysis of C/ebpβ, Fabp4, and Pparg mRNA expressions in BMSCs from control, Vhl cKO, and Vhl/Usp26 dKO mice after different days (0, 4, and 8 days) of adipogenic differentiation. n = 3 in each group. U) Representative images of alcian blue staining of BMSCs from control, Vhl cKO, and Vhl/Usp26 dKO mice after 7 days of chondrogenic differentiation. n = 6 in each group. Scale bar, 50 µm. V) qPCR analysis of Aggrecan, Col2a1, and Sox9 mRNA expressions in BMSCs from control, Vhl cKO, and Vhl/Usp26 dKO mice after different days (0, 3, and 7 days) of chondrogenic differentiation. n = 3 in each group. Mice age in A) to H), 6‐month‐old. Mice age in I) to V), 18‐month‐old. BMSCs from mice at P5 were used in I)‐V). Data are represented as mean ± SD. Statistical significance was determined by one‐way ANOVA in B), D), G), H), I), J), K), L), N), P), and S), two‐way ANOVA in Q), T), and V). *p < 0.05, **p < 0.01, ***p < 0.001.

Having observed a decrease in the expression of USP26 in elderly BMSCs resulting from reduced HIF‐1α expression, we next aimed to investigate whether activating the HIF‐1α pathway could combat aging and improve the self‐renewal and multipotent differentiation of aged BMSCs by driving USP26 expression. We genetically manipulated BMSCs by deleting Vhl through the crossing of Prx1‐Cre and Vhl^flox/flox^ mice, resulting in the activation of HIF‐1α in BMSCs (referred to as Vhl cKO). Subsequently, the Prx1‐Cre; Vhl^flox/flox^; Usp26^flox/flox^ (referred to as Vhl/Usp26 dKO) mice were generated by crossing Vhl cKO mice with Usp26^flox/flox^ mice, thus simultaneously activating HIF‐1α and deleting Usp26 in BMSCs in vivo. Both Vhl cKO mice and Vhl/Usp26 dKO mice were viable and were born at the expected Mendelian ratio. The results, as shown in **Figure** **10**A,B, indicate that Vhl cKO significantly increased the protein expression of HIF‐1α in femoral tissues and the gene expression of Usp26 in bone marrow cavity BMSCs. In contrast, Vhl/Usp26 dKO significantly inhibited the upregulation of USP26 induced by Vhl cKO. Consistent with previous reports, Vhl cKO mice exhibited a lean phenotype,^[^ ^35^ ^]^ as depicted in Figure S11A,B (Supporting Information), with significantly lower body weights compared to control mice at 6 months of age. However, Usp26 deletion led to a noticeable increase in the body weight in Vhl cKO mice.

Previous studies have shown that knocking out Vhl in osteoblast precursors leads to significant accumulation of bone mass in the bone marrow cavity.^[^ ^36^ ^]^ Micro‐CT analysis revealed that, compared to Vhl^flox/flox^ (WT) mice, knockout of Vhl in BMSCs also led to a significant increase in intracellular bone mass generation, narrowing of the bone marrow cavity, and significant increases in bone trabecular parameters including BV/TV, Tb.N, and Tb.Th, with a significant decrease in Tb.Sp. However, in Vhl/Usp26 dKO mice, knocking out Usp26 reduced the bone mass accumulation caused by Vhl knockout, thereby alleviating bone mass deposition (Figure 10C,D). H&E staining of the femurs of WT, Vhl cKO, and Vhl/Usp26 dKO mice showed similar results (Figure 10E). IHC analysis for FABP4 revealed a significant increase in FABP4‐positive cells in Vhl cKO mice, while demonstrating a decrease in FABP4‐positive cells in Vhl/Usp26 dKO mice (Figure 10F,G). Consistently, the adipocyte area/tissue area significantly increased in Vhl cKO mice, but decreased in Vhl/Usp26 dKO mice (Figure 10H).

The HIF‐1α pathway is known to regulate the aging and differentiation processes of BMSCs.^[^ ^37^ ^]^ In order to further investigate the role of USP26 as a downstream molecule of HIF‐1α in the regulation of aging and age‐related self‐renewal and multi‐lineage differentiation declines of BMSCs, we extracted BMSCs from Vhl cKO, Vhl/Usp26 dKO, and control mice that were 18 months old. The results in Figure 10I,J show that Vhl cKO significantly decreased the expression of markers associated with aging in BMSCs, as indicated by SA‐β‐Gal staining and the gene expression of P16 and P21. However, this decrease was significantly inhibited by Usp26 cKO. The results from the EdU staining and CFU assay indicated that the activation of the HIF‐1α pathway significantly increased the proliferation of aged BMSCs. However, this effect was significantly suppressed by Usp26 deletion (Figure 10K,L). The expression of multipotency‐related protein and SIRT2 revealed that HIF‐1α activation by Vhl deletion significantly enhanced the expression of SIRT2 and the multipotent capacity of aged BMSCs. However, this enhancement was inhibited by the knockdown of USP26. (Figure 10M,N). Furthermore, the results of osteogenic, adipogenic, and chondrogenic differentiation showed that the activation of HIF‐1α by Vhl deletion significantly promoted the differentiation potential of aged BMSCs, while knockout of Usp26 significantly inhibited this process (Figure 10O–V). In conclusion, our results demonstrated that the activation of the HIF‐1α‐USP26 pathway combated aging and improved the self‐renewal and multipotent differentiation of aged BMSCs.

## 3 Discussion

**Figure 11:**
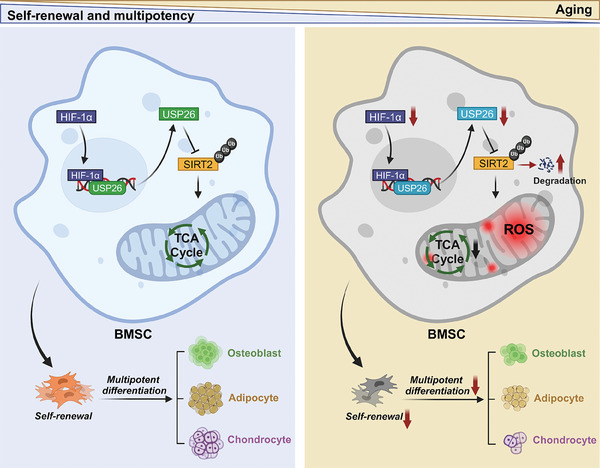
Schematic of the HIF‐1α/USP26/SIRT2 axis combats age‐related declines in self‐renewal and multipotent differentiation of BMSC by maintaining mitochondrial homeostasis. As BMSCs age, decreased HIF‐1α expression reduces its binding to the −191— −198 and −262— −269 bp on the USP26 promoter, thereby dampening Usp26 transcriptional expression. This leads to reduced protein expression of SIRT2 through the promotion of its ubiquitination and degradation. Consequently, mitochondrial dysfunction occurs in BMSCs, resulting in senescence phenotypes characterized by decreased self‐renewal and impaired multilineage differentiation potentials.

Stem cell aging is a prominent theory of organismal aging.^[^ ^38^ ^]^ For decades, BMSCs have been considered as an excellent source for stem cell‐based therapy in anti‐aging treatment, owning to their exceptional clinical characteristics.^[^ ^1a^ ^]^ These characteristics include easy accessibility, simplicity of isolation, self‐renewal and proliferation abilities, multilineage differentiation potentials, and immunomodulatory effects.^[^ ^2c,d^ ^]^ However, numerous studies have shown that BMSCs undergo functional deterioration and gradually lose their multipotency with age in vivo or through prolonged culture in vitro, which hinders their therapeutic potential.^[^ ^1b^ ^]^ Therefore, it is crucial to uncover the mechanisms behind BMSCs senescence to develop innovative techniques for combating aging and age‐related self‐renewal and multipotency declines of BMSCs. Here, we discovered that decreased USP26 expression was correlated with aging and a reduction in the self‐renewal and multilineage differentiation potentials of BMSCs. Mechanistically, the decreased HIF‐1α expression in aging BMSCs leads to reduced transcriptional expression of Usp26, resulting in decreased protein expression of SIRT2 due to its ubiquitination degradation. This, in turn, leads to mitochondrial dysfunction in BMSCs, ultimately resulting in senescence phenotypes such as decreased self‐renewal and impaired multilineage differentiation potentials (**Figure** **11**).

Research on USP26 has revealed its involvement in various biological processes. Numerous studies have investigated the role of USP26 in male fertility and spermatogenesis owing to its high expression in the testes and its participation in spermatid development.^[^ ^39^ ^]^ Moreover, USP26 has been found to interact with receptor‐associated protein 80, a DNA damage response protein, indicating a potential role in the repair of damaged DNA and maintenance of genomic stability.^[^ ^40^ ^]^ Additionally, USP26 enhances esophageal squamous cell carcinoma metastasis by stabilizing Snail,^[^ ^41^ ^]^ and it is essential for embryonic stem cell differentiation by stabilizing PRC1 complex components.^[^ ^42^ ^]^ In our previous study, we established that USP26 acts as a regulator of bone homeostasis, showcasing osteoprotective properties by orchestrating bone formation and resorption.^[^ ^9^ ^]^ Building upon this, herein we have discovered that USP26 levels decline in both mice and human BMSCs as they age. However, by reintroducing USP26 into aged mouse BMSCs, we were able to effectively combat the effects of aging and age‐related declines in self‐renewal and multipotency.

Mitochondria are the powerhouses of the cell, responsible for generating ATP through oxidative phosphorylation.^[^ ^43^ ^]^ As cells age, mitochondrial function declines, which has been increasingly recognized as a key factor in the aging‐related declines of self‐renewal and multipotency of BMSCs.^[^ ^2d^ ^]^ A study by Zheng et al. (2020) found that aged BMSCs exhibited lower mitochondrial membrane potential, decreased ATP production, and increased oxidative stress compared to young BMSCs.^[^ ^44^ ^]^ Enhancing mitochondrial function in aged BMSCs through the use of mitochondrial‐targeted antioxidants improved the proliferative capacity and differentiation potential of aged BMSCs.^[^ ^45^ ^]^ In our study, we observed that the mitochondrial morphology in Usp26‐deficient cells were swollen, fragmented, with reduced or absent cristae, accumulation of superoxide within the mitochondria, oxidative stress, decreased or lost membrane potential, and severe impairment of mitochondrial respiratory function. Interestingly, reintroducing Usp26 expression in aging BMSCs significantly improved mitochondrial morphology and respiratory function. Considering that reintroducing Usp26 can significantly decrease aging indicators in BMSCs and improve their self‐renewal and differentiation potential, it is reasonable to conclude that USP26 regulates the senescence and multi‐lineage differentiation of BMSCs by affecting mitochondrial function.

As a deubiquitinating enzyme, USP26 is involved in various biological processes by maintaining the stability of target proteins through its deubiquitination activity.^[^ ^9^, ^41^, ^42^ ^]^ Through the analysis of differential proteins in Usp26 cKO and its control BMSCs, as well as protein immunoprecipitation analysis of USP26 with SIRT2, and gain‐ or loss‐of‐function experiments with SIRT2, we further confirmed that SIRT2 is responsible for the dysregulated mitochondrial function, aging, and impaired self‐renewal and multipotency of Usp26 cKO BMSCs. However, it must be emphasized that it is currently unknown whether other proteins are also involved in the regulation of mitochondrial function, self‐renewal, and multipotent differentiation of BMSCs by USP26, as more than 2 000 proteins were found to be differentially expressed between Usp26 cKO BMSCs and their controls.

The bone marrow niche is a complex microenvironment that regulates the behavior of various resident cells, including hematopoietic stem cells (HSCs) and MSCs.^[^ ^14^ ^]^ During aging, significant changes occur in the bone marrow niche, leading to alterations in hematopoiesis and bone metabolism.^[^ ^46^ ^]^ The bone marrow environment is characterized by hypoxic conditions, and aging‐related vascular changes and altered oxygen levels can impact HIF‐1α signaling in the bone marrow niche, affecting stem cell function and tissue regeneration.^[^ ^47^ ^]^ Research indicates that hypoxia/HIF‐1α activation can enhance cell proliferation rates, maintain stem cell properties, inhibit senescence, and improve the differentiation ability of BMSCs.^[^ ^48^ ^]^ This study further reveals that HIF‐1α controls aging and age‐related declines in self‐renewal and multipotent differentiation of BMSCs through USP26. Specifically, HIF‐1α drives Usp26 transcriptional expression by increasing Usp26 promoter activity through binding to the −191 — −198 and −262 — ‐269 bp on the Usp26 promoter.

It is important to note that the HIF‐1α pathway primarily responds to changes in oxygen levels.^[^ ^49^ ^]^ In addition to HIF‐1α, multiple signaling pathways such as Wnt and Notch are crucial for maintaining and regulating the stem cell population in the bone marrow niche during aging.^[^ ^50^ ^]^ Canonical Wnt/β‐catenin signaling tends to decrease with age and plays a major role in regulating bone turnover, directly linked to osteoporosis in the elderly.^[^ ^51^ ^]^ A decrease in the Wnt/β‐catenin pathway in BMSCs can lead to increased adipogenic differentiation abilities,^[^ ^52^ ^]^ which is in contrast to our findings of decreased adipogenic differentiation abilities in Usp26 cKO BMSCs. However, considering our previous research showing that USP26 stabilizes β‐catenin to promote the osteogenic activity of BMSCs,^[^ ^9^ ^]^ it cannot be ruled out that USP26 may regulate the osteogenic differentiation of BMSCs through β‐catenin. Furthermore, notch signaling also becomes aberrant with aging, specifically influencing the balance of renewal and differentiation in HSCs, impacting both hematopoietic and immune system aging.^[^ ^53^ ^]^ It is important to emphasize that these pathways interact with each other to ensure the proper functioning of the bone marrow niche. Notch can influence Wnt signaling,^[^ ^54^ ^]^ and changes in oxygen levels regulated by HIF‐1α can affect Wnt and Notch pathway activities.^[^ ^55^ ^]^ Thus, understanding the distinct and overlapping contributions of these pathways is crucial for regulating the aging bone marrow niche and impacting various cellular functions and tissue homeostasis.

In conclusion, our study provides evidence that the impaired HIF‐1α/USP26/SIRT2 axis in BMSCs leads to mitochondrial dysfunction, ultimately resulting in the aging and age‐related declines in self‐renewal and multipotency of BMSCs. Therefore, the identification of USP26 as correlated with aging in BMSCs, along with its expression and action mechanisms, suggests that USP26 represents a novel therapeutic target for combating the aging and age‐related declines in self‐renewal and multipotency of BMSCs.

## 4 Experimental Section

### Ethics Statement

Human BMSCs were isolated from bone marrow biopsies obtained from 15 patients who underwent UKA surgery for end‐stage OA at Ruijin Hospital, Shanghai Jiao Tong University School of Medicine. A total of 5–10 mL of tibial bone marrow was collected using Bone Marrow Prep Syringes (Pharmacy Department, NIH, Bethesda, MD) and then washed with 2.5x volume of HBSS.^[^ ^56^ ^]^ Cells expressing the typical surface markers of BMSCs (CD45^−^, CD31^−^, CD90^+^, and CD105^+^) were collected for subsequent analysis.^[^ ^57^ ^]^ All participants signed a written informed consent prior to enrollment (Reference number: 1.0/2020‐6‐29). All animal experiments were conducted in accordance with the protocol approved by the Shanghai Jiao Tong University Animal Care and Use Committee and directly in accordance with the guidelines of the Ministry of Science and Technology of China on Animal Care (Reference number: SYXK (Shanghai) 2018‐0027).

### Transgenic Mouse Model

Usp26 floxed mice were provided by GemPharmatech Co., Ltd. (Nanjing, China). The mice were constructed as follows: First, two sgRNAs were constructed and transcribed in vitro to target the introns on both sides of the floxed region of Usp26. In addition, a donor vector with the loxp fragment was designed and constructed in vitro. Following this, Cas9 mRNA, sgRNA, and the donor were coinjected into zygotes. The zygotes were then transferred into the oviduct of pseudopregnant ICR females at 0.5 dpc. F0 mice were born after 19–21 days of transplantation. All offspring of the ICR female mice (F0 mice) were identified by Polymerase chain reaction (PCR) and sequencing of tail DNA, and positive F0 mice were crossed with C57BL/6J mice to produce heterozygous mice.

Prx1‐Cre mice were purchased from the Jackson Laboratory (JAX Stock, #005584, USA); Vhl^flox/flox^ mice were kindly provided by Dr. Thomas L. Clemens (Department of Orthopedic Surgery, John Hopkins University School of Medicine, Baltimore, MD).

Usp26^flox/flox^ mice were crossed with Prx1‐Cre mice to generate Usp26 conditional knockout mice in BMSCs (Prx1‐Cre;Usp26^flox/flox^) and littermate controls. To establish Vhl conditional knockout mice in BMSCs, Vhl^flox/flox^ mice were crossed with Prx1‐Cre mice to generate Vhl conditional knockout mice in BMSCs (Prx1‐Cre;Vhl^flox/flox^) and littermate controls. To establish Vhl/Usp26 double knockout mice in BMSCs (Vhl/Usp26 dKO), Vhl cKO mice were crossed with Usp26^flox/flox^. All mice were on a C57BL/6 background. All mice were housed in pathogen‐free animal facilities under 12 h light and 12 h dark cycle at Ruijin Hospital, Shanghai Jiao Tong University School of Medicine, Shanghai, China. All mice were followed by genotyping with One Step Mouse Genotyping Kit for mouse Tail (Vazyme, #PD101, China). PCR genotyping primers are listed in Table S1 (Supporting Information).

### Knee Cartilage Defects Models

Surgery of knee cartilage defects models was performed as previously reported.^[^ ^58^ ^]^ According to the different degree of cartilage defect, osteochondral defects model and full‐thickness cartilage defects were constructed respectively. In the osteochondral defect model, eight‐week‐old Usp26 cKO mice and the littermate controls were intraperitoneally injected with pentobarbital sodium. 0.6 mm diameter cylinder‐shape osteochondral defect at the trochlear groove on the knee joint was operated with a trephine (Komet, Germany) by media parapatellar approach. The osteochondral defect penetrated the bone marrow, allowing the BMSCs to be recruited to the defect area. The skin was sutured and bone samples were collected 7 and 14 days later for Micro‐CT analysis and histological analysis. In cases of full‐thickness cartilage defects, 0.6 mm diameter cylinder‐shaped full‐thickness cartilage defects were created, and the defects were localized to the cartilage layer. Sulfated hyaluronic acid (SHA) hydrogel is commonly utilized as a carrier to transport stem cells for the treatment of cartilage defects, as it exhibits excellent biocompatibility and degradation properties.^[^ ^59^ ^]^ In the control group, the defects were filled with 10 µl of blank hydrogel. In the other groups, the defects were filled with either 10 µl of hydrogel containing Control BMSCs or 10 µl of hydrogel containing USP26 overexpression BMSCs, with BMSCs suspended in the hydrogel at a density of 5 × 10^7^ cells/mL. Subsequently, the skin was sutured, and bone samples were collected 7 and 14 days after post‐surgery for Micro‐CT and histological analysis (n = 6).

### Mouse BMSCs Isolation and Identification

To isolate BMSCs for next experiments, the femur and tibia from different mice, including 2‐month‐old mice, 15‐month‐old mice, 20‐month‐old mice, Usp26 cKO mice, Vhl cKO mice, Vhl/Usp26 dKO mice, and the corresponding littermate controls, were collected. Cells from bone marrow were flushed with α‐MEM through the femur and tibia with 1 mL injection syringe. The cells were expanded using the mouse MesenCult Proliferation Kit (STEMCELL Technologies).^[^ ^60^ ^]^ BMSCs from passages three to five were used in this study. In this context, FACS analysis was performed to determine the purity of primary BMSCs of P3. In flow cytometry analysis, cell fragments and adherent cells through scattered light channels (FSC/SSC) were excluded, and living cell populations were gated using DAPI (Figure S1A, Supporting Information). Within the gate of living cells, most of the cells were identified as CD45^−^CD31^−^(99.8%) while they were CD44^+^ (93.3%) and CD105^+^(92.4%) (Figure S1B, Supporting Information). In addition, the cells showed a classic spindle or triangle shape under the light microscope (Figure S1C, Supporting Information). Further functional assays revealed significant potential for osteogenic, adipogenic, and chondrogenic differentiation, as indicated by ARS and ALP staining, Oil red O staining, and Alcian blue staining, respectively (Figure S1D‐F, Supporting Information).

### EdU Staining

For the analysis of BMSCs proliferation, cells were cultured in a 24‐well plate (1.2 × 10^4^‐2 × 10^4^) for 24 h. EdU working liquid preheated at 37 °C was added to the 24‐well plate in equal volume for 2 h under the instructions of assay (Beyotime, C0071S, China). Then cells were fixed with 4% paraformaldehyde (Santa cruz, #281692, China) for 25 min at room temperature and washed with PBS for 3 min three times. Afterward, cells were permeabilized with 0.3% Triton in PBS for 15 min and washed with PBS for 3 min three times again. The cells were incubated with 1.5 ug mL⁻^1^ DAPI solution and counterstained with DAPI (Sigma‐Aldrich, D9542, USA). The images were obtained by fluorescence confocal microscope (ZEISS, Germany), and the number of EdU positive cells was calculated using the ImageJ software.

### Colony‐Formation Assay

For serial colony formation assay, 3 × 10^3^ BMSCs were plated in a 6‐well plate and cultured with 2 ml MesenCult™ Expansion Medium (STEMCELL Technologies).^[^ ^60^ ^]^ The cells were processed with crystal violet staining after 7 days of culture or upon the 2nd plating. For the 2nd plating, colonies were dissociated into a single‐cell suspension with DPBS, washed three times, and 3 × 10^3^ cells were re‐plated in a 6‐well plate with 2 mL MesenCult™ Expansion Medium and cultured for another 7 days. Subsequently, crystal violet staining was performed, followed by the 3rd, 4th, and 5th re‐plating according to the 2nd plating described. Colonies containing 50 or more cells were counted.

### Quantitative Real‐Time PCR (qRT‐PCR) Assay

TRIzol reagent (Invitrogen, #15596026CN, USA) was used to extract total RNA from the bone tissue or cells treated with DNase I (Invitrogen, AM2222) to remove genomic DNA. NanoDrop microspectrophotometer (Thermo Fisher, USA) was employed to calculate the RNA quantity and purity. Furthermore, 1 µg of total RNA sample (260/280 ≥ 1.8) was used to obtain cDNA via Prime‐Script RT reagent assay (Vazyme, R333, China). Quantitative RT‐PCR was performed to amplify cDNA using SYBER Green PCR Master Mix (Takara Biotechnology, RR036A, Japan). β‐actin was quantified as an endogenous control of different mRNA expression. Lists of primers were described in Table S2 (Supporting Information).

### Western Blotting and Co‐immunoprecipitation (Co‐IP)

RIPA lysis buffer (Beyotime, P0013B, China) was used to collect proteins from bone and cell samples on ice with protease inhibitors (Thermo Fisher, #78442, USA). The supernatant was collected after centrifugation at 14000 × g. BCA Protein Assay kit (Thermo Fisher, #23227, USA) was used determine the protein concentration under the guidance of manufacturer's instructions. The protein were denatured after high heat for 10 min. 8–20% SDS‐PAGE gels were used to separate different proteins, which were transferred onto PVDF membranes (Merck, #05317, Germany). The membranes were blocked with 1% BSA in TBS‐T (0.1% Tween‐20) for 1 h at room temperature and incubated overnight at 4 °C with primary antibodies. Lists of antibodies were described in Table S3 (Supporting Information). The membranes were washed with TBS‐T for 10 min three times and soaked in secondary antibodies for 1 h. The membranes were washed with TBS‐T for 10 min three times and detected by enhanced chemiluminescence reagent (Thermo Fisher, #35055, USA). β‐actin was quantified for normalization.

For Co‐IP, whole cell extracts were prepared and incubated with the corresponding antibodies at 4 °C overnight. After addition of the protein A&G beads (Abmart), the incubation was continued at 4 °C for 6 h. The co‐precipitated proteins were washed, eluted at 95° C for 5 min with SDS‐loading buffer (Beyotime, P0013G, China), and then analyzed by western blotting.

### Alkaline Phosphatase Staining and Alizarin Red S Staining

BMSCs were isolated from different mice and cultured in 12‐well plates in the mouse osteogenic differentiation medium (Cyagen, MUXMX‐90021, China). After 14 days of induced differentiation, the cells were fixed with 4% paraformaldehyde for 20 min at room temperature and washed with PBS for 3 min. Cells were performed Alkaline phosphatase staining and Alizarin red s staining by Alkaline Phosphatase Color Development Kit (Beyotime, P0321S, China), and Alizarin Red S Solution (Cyagen, OILR‐10001, China) under the guidance of manufacturer's instructions. The images were obtained with digital camera (Canon, Japan).

### Oil Red O Staining

BMSCs were isolated from different mice and cultured in 6‐well plates with mouse adipogenic differentiation medium (Cyagen, MUXMX‐90031, China). After 21 days of induced differentiation, cells were fixed with 4% paraformaldehyde for 20 min at room temperature and washed with PBS for 3 min. Cells were performed Oil red O staining by Oil Red O staining kit (Kingmorn, FS0404R, China) under the guidance of manufacturer's instructions. The images were obtained with digital camera (Canon, Japan).

### Alcian Blue Staining

BMSCs were isolated from different mice and cultured in 6‐well plate with mouse chondrogenic differentiation medium (Cyagen, MUXMX‐90041, China). After 7 days of induced differentiation, the cells were fixed with 4% paraformaldehyde for 20 min at room temperature and washed with PBS for 3 min. Alcian blue staining was performed by Alcian blue staining kit (Cyagen, ALCB‐10001, China) under the guidance of manufacturer's instructions. The images were obtained with digital camera (Canon, Japan).

### Micro‐CT Analysis

As mentioned previously, Micro‐CT analysis of the left femur of each mouse was performed.^[^ ^9^ ^]^ The femurs were fixed in 4% paraformaldehyde and scanned by a Skyscan 1172 (Aartselaar, Belgium) with a 10‐µm misotropic voxel size, 50 keV, 500 µA, and 0.7° rotation step, under the guidelines of the American Society for Bone and Mineral Research (ASBMR).^[^ ^61^ ^]^ Based on the trabecular and cortical parameters, a specific area was defined as regions of interest (ROIs). The ROI of the trabecula extends 1 mm from the proximal end of the backbone to the distal end of the growth plate. The ROI of the cortex was extended 3 mm from the proximal end of the shaft to the distal end of the growth plate. 2D cross sections of the bone trabeculae and cortex were scanned for 3D reconstruction. Trabecular bone parameters were measured including BMD, BV/TV, Tb.Th, Tb.N, and Tb.Sp. Cortical bone parameters were measured including BMD, BV/TV, and Ct. Th. In order to further examine osteochondral defect formation in the knee joint, the bone defect site was defined as the volume of interest (VOI). BMD, BV/TV, Tb.Th, and Tb.Sp were calculated within the VOI.

### Bone Histomorphometry and Immunohistochemistry

The femur was fixed with 4% paraformaldehyde for 48 h and decalcified with EDTA solution (Servicebio, G1150, China) for 3 weeks to prepare paraffin embedded sections. To section the femur and knee joints, sagittal sections were taken from the lateral condyle of the femur. H&E staining was performed every 5 sections under the guidance of manufacturer's instructions. For IL‐6, FABP4, and SIRT2 immunohistochemistry of the femurs, paraffin embedded sections were dewaxed to water. After antigen retrieval, the slides were washed by PBS for 10 min three times. 3% hydrogen peroxide solution was used to block endogenous peroxidase. The sections were blocked with 3% BSA for 1 h at room temperature and incubated overnight at 4 °C with IL‐6 (Abcam, ab290735, China), FABP4 (Abcam, ab92501, China), SIRT2 (Proteintech, 19655‐1‐AP, China) and primary antibodies. The sections were washed with PBS for 10 min three times and soaked in secondary antibodies for an hour. After washed with PBS for 10 min three times, the sections were colored with freshly prepared DAB solution.

For the bone histomorphometry of the whole skeleton of control and cKO embryos at E16.5, the femurs were fixed with 4% paraformaldehyde for 48 h and sectioned without decalcification. H&E staining and Alcian red staining of the femurs were performed every five sections under the guidance of the manufacturer's instructions.

### Skeletal Staining

Whole bones of embryonic mice were stained with Alizarin red S and Alcian blue as reported.^[^ ^62^ ^]^ Embryonic mice were collected, and the skin and internal organs were removed clearly. The remaining embryonic mouse skeleton were fixed in 95% EtOH for 24 h. Cartilage of embryonic mice was stained by Alcian blue solution (0.3% in 80% EtOH and 20% Acetic Acid), and the bone of embryonic mice were stained by Alizarin Red stain (0.1% in 90% EtOH and 10% Acetic Acid) for 3 days at room temperature. After rinsing with water, the samples were placed in 1% KOH for 1 day and then in the removal solution (75:25 ratio of 1% KOH to glycerin) for 3 days until the skeleton was clearly visible. The skeletons of embryonic mice were preserved in a 50:50 ratio of 90% ethanol and 75% glycerol.

### Senescence‐Associated β‐Galactosidase (SA‐β‐Gal) Staining

After plated in 12‐well plate at a density of 2.5 × 10^4^‐5 × 10^4^ per well, BMSCs were fixed with 4% paraformaldehyde for 20 min at room temperature and washed with PBS for 1 min. Cells were incubated with β‐galactosidase staining solution (Beyotime, C0602, China) overnight at 37° in the dark. After staining, the cells were washed with PBS for 1 min and the representative images were obtained under the microscope (ZEISS, Germany). The number of SA‐β‐gal positive cells was calculated using the ImageJ software.

### Enzyme‐Linked Immunosorbent Assays (ELISA) Assay

Fresh mouse femur specimens were collected and the bone marrow was flushed by α‐MEM culture medium. The bone marrow samples were centrifuged at 300 g for 5 min to remove cell fragments and the supernatant was obtained. The concentrations of TNF‐α, IL‐1, IL‐6, MMP3, and MMP13 were tested under the guidance of the manufacturer's instructions by following Elisa Kits: TNF‐α (Abcam, ab208348, China), IL‐1 (Abcam, ab199076, China), IL‐6 (Abcam, ab222503, China), MMP3 (Abcam, ab203363, China), and MMP13 (Elabscience, E‐EL‐M0076, China).

### Immunofluorescent Staining

BMSCs were cultured in Nunc petri dish on a glass bottom (Thermo Fisher, #150682, USA) at a density of 0.5 × 10^4^‐1 × 10^4^ per well. Then cells were fixed with 4% paraformaldehyde for 25 min at room temperature and washed with PBS for 1 min three times. Afterward, cells were permeabilized with 0.1% Triton in PBS for 10 min. After washed with PBS for 1 min three times, cells were blocked with 1% BSA in PBS for 1 h at room temperature and incubated overnight at 4 °C with γ‐H2AX primary antibodies (Abcam, ab81299, China). Then the cells were washed with PBS for 1 min three times and incubated with secondary antibodies for 1 h. After washed with PBS for 1 min three times, the cells were incubated with 1.5 ug mL⁻^1^ DAPI solution and counterstained with DAPI. The images were obtained by fluorescence confocal microscopy (ZEISS, Germany) and the number of γ‐H2AX positive cells were calculated using the ImageJ software.

### RNA Sequencing

BMSCs total RNA extraction, mRNA library construction and sequencing were performed as reported previously.^[^ ^9^ ^]^ After the final transcriptome was generated, the FPKM was calculated using StringTie and ballgown to estimate significant differentially expressed genes (DEGs) by R package edgeR. KEGG pathway analysis, GO analysis, and GSEA were performed to detect different pathways between BMSCS from control and Usp26 cKO mice.

TEM was performed as previously reported.^[^ ^63^ ^]^ Cells were collected by TrypLE™ Express Enzyme (Thermo Fisher, #12605010, USA). After pelleted by centrifugation at 500 × g for 5 min, cells were fixed with glutaraldehyde at 4 °C overnight. Dehydration was then completed by a graded series of ethanol, which was then wetted in the mixture of acetone and SPI‐PON812 resin. Ultrathin sections were acquired by Leica EM UC6 microtome and then stained with uranyl acetate and lead citrate. Transmission Electron Microscope (FEI Company, USA) was used to capture the images at 100 kV. Next, the number of abnormal mitochondria in each sample was calculated to determine the relative percentage of abnormal mitochondria. The definition of abnormal mitochondria follows previous reports.^[^ ^63^ ^]^ Briefly, the mitochondria with obvious damage of mitochondrial ridge structure and vesicles in mitochondrial inner membrane were defined as abnormal mitochondria. The number of abnormal mitochondria and total mitochondria were measured by ImageJ.

### Mitochondrial oxygen consumption rates (OCR) Measurement

OCR measurement was performed as previously reported.^[^ ^64^ ^]^ BMSCs from different mice were plated in XF‐24‐well culture microplates at density of 1.5 × 10^4^‐2.0 × 10^4^ per well, which was used to measure the oxygen consumption by extracellular analyzer (Seahorse Bioscience, USA). The Seahorse XF sensor cartridge was incubated by calibration solution at 37 °C incubator without CO_2_ overnight. Seahorse assay medium was kept 37 °C by water bath preheated. XF‐24‐well culture microplates were washed by Seahorse assay medium twice and incubated at 37 °C incubator without CO_2_ for 1 h with 180 µL Seahorse assay medium per well. Respiration regulators oligomycin (1 µmol L⁻^1^), FCCP (1 µmol L⁻^1^), and Rotenone/Antimycin A (0.5 µmol L⁻^1^) were prepared and added to the pre‐calibrated Seahorse XF sensor cartridges. The experiment was run and analyzed by software “Wave”. The OCR values were calculated including basal respiration, maximal respiration capacity, spare respiration capacity, and ATP production.

### Liquid Chromatography‐Mass Spectrometry (LC‐MS) Analysis

RIPA lysis buffer (Beyotime, P0013B, China) was used to collect proteins from BMSCs in control and Usp26 cKO mice. The amount of protein was quantified with the BCA Protein Assay Kit (Thermo Fisher, #23227, USA). Protein digestion by trypsin was performed according to filter‐aided sample preparation (FASP) procedure described by Matthias Mann. The digest peptides of each sample were desalted on C18 Cartridges (Empore SPE Cartridges C18 (standard density), bed I.D. 7 mm, volume 3 mL, Sigma), concentrated by vacuum centrifugation and reconstituted in 40 µL of 0.1% (v/v) formic acid. DTT (with the final concentration of 40 mM) was added to each sample, respectively, and mixed at 600 rpm for 1.5 h (37 °C). After the samples cooled to room temperature, IAA was added with the final concentration of 20 mM into the mixture to block reduced cysteine residues, and the samples were incubated for 30 min in darkness. Next, the samples were transferred to the filters respectively. The filters were washed with 100 µL UA buffer three times and then 100 µL 25 mM NH_4_HCO_3_ buffer twice. Finally, trypsin was added to the samples (the trypsin: protein (wt/wt) ratio was 1:50) and incubated at 37 °C for 15–18 h (overnight), and the resulting peptides were collected as a filtrate. The peptides of each sample were desalted on C18 Cartridges (Empore SPE Cartridges C18 (standard density), bed I.D. 7 mm, volume 3 mL, Sigma), concentrated by vacuum centrifugation and reconstituted in 40 µL of 0.1% (v/v) formic acid. The peptide content was estimated by UV light spectral density at 280 nm using an extinction coefficient of 1.1 of 0.1% (g L⁻^1^) solution, which was calculated on the basis of the frequency of tryptophan and tyrosine in vertebrate proteins. LC‐MS/MS analysis was performed on a timsTOF Pro mass spectrometry (Bruker) that was coupled to Nanoelute (Bruker). The peptides were loaded onto a C18‐reversed phase analytical column (Thermo Scientific Easy Column, 25 cm long, 75 µm inner diameter, 1.9 µm resin) in 95% buffer A (0.1% Formic acid in water) and separated with a linear gradient of buffer B (99.9% acetonitrile and 0.1% Formic acid) at a flow rate of 300 nL/min. The mass spectrometer was operated in positive ion mode. The electrospray voltage applied was 1.5 kV. Precursors and fragments were analyzed at the TOF detector over a mass range of m/z 100–1700. The timsTOF Pro was operated in parallel accumulation serial fragmentation (PASEF) mode, PASEF mode data collection was performed based on the following parameters: Ion mobility coefficient (1/K0) value was set from 0.6 to 1.6 Vs cm2; 1 MS and 10 MS/MS PASEF scans. Active exclusion was enabled with a release time of 24 s. MS raw data for each sample were combined and searched using the MaxQuant 1.6.14 software for identification and quantitation analysis.

### Lentivirus Interfering RNA (Usp26, Sirt2 Overexpression)

For gene overexpression, mouse Usp26 or Sirt2 was cloned into a lentiviral vector backbone‐pLV[Exp]‐EGFP:T2A:Puro‐EF1A via Golden Gate method,^[^ ^65^ ^]^ and mCherry was also inserted into the same vector backbone to make a negative control. Sanger sequencing and restriction enzyme digestion tests were used to verify all vectors at the last step of vector construction. Overexpression vectors of Usp26 or Sirt2 were co‐transfected with pLV/helpr‐SL3 (gag/pol element), pLV/helper‐SL4 (pRev element) and pLV/helper‐SL5 (pVSVG element), by calcium phosphate transfection method, into HEK293T cells. 48 h after transfection, supernatant carrying the lentiviral particles was collected for concentration and purification to prepare the final lentivirus for transduction, and the titers were confirmed by Lenti‐X p24 Rapid Titer Kit (Clontech. #632200, China).

### Tissue Dissociation for 10x Genomics

Ten male C57BL/6J mice, consisting of five aged 2 months and five aged 20 months, were utilized to conduct scRNA‐seq analysis. Bone marrow cells were harvested by flushing the tibiae and femurs of the mice with α‐MEM mixed with 2% fetal bovine serum. The dissociated cells were filtered through a 40 µm filter, centrifuged at 1500 rpm for 5 min, and then incubated for 5 min at room temperature in ACK lysis buffer for red blood cell lysis. Neutralization was achieved by adding 20 mL of cell suspension medium. The cells were sorted into catching medium (0.04% BSA in PBS), and single‐cell samples were counted by using a hemocytometer with trypan blue and Countess™ II Automated Cell Counter. A total of 20 069 bone marrow cells were used for scRNA seq analysis, including 11 066 cells from 2‐month‐old mice and 9003 cells from 20‐month‐old mice. When cell viability was greater than 95%, the cells were loaded onto a 10x Genomics Chromium chip following the manufacturer's instructions.

### Single‐Cell cDNA Library Preparation and Sequencing

The Single Cell 3′ Library was primarily constructed using the Chromium Single Cell 3′ Reagent Kits User Guide (v2 Chemistry). First, a gel bead emulsion (GEM) was generated by mixing single cell suspension, gel beads, and oils on the 10x Genomics chromium controller. After droplet formation, the samples were applied to PCR tubes, and reverse transcription was carried out using a T100 Thermal Cycler (Bio‐Rad). The reverse transcription involved incubation at 53 °C for 45 min, followed by 85 °C for 5 min and then preservation at 4 °C. The cDNA was synthesized, amplified, and subjected to quality evaluation by an Agilent Bioanalyzer 2100. To construct the library, the P5 primer, Read 2 (a primer site for sequencing read 2), Sample Index, and P7 primer were added in sequence. The resulting library underwent quality control before being sequenced using the Illumina HiSeq4000 PE125.

### Processing of Single‐Cell RNA‐Seq Data and Quality Control

Cell Ranger (version 5.0) was utilized to eliminate low‐quality reads, align reads to the human reference genome (GRCh38), assign cell barcodes, and generate unique molecular identifier (UMI) matrices. The resulting gene expression matrices were analyzed using R software (version 3.6.1) and the Seurat package (version 3.2.0). All samples were combined into one Seurat object using the “merge” function in Seurat. Next, cells with fewer than 500 genes detected or >10% mitochondrial UMI counts were filtered out.

### Dimension Reduction, Unsupervised Clustering, and Cell‐Type Annotation

Dimension reduction and unsupervised clustering were performed using the standard workflow in Seurat. The Find Variable Features function was used to identify highly variable genes (HVGs) in the single‐cell gene expression data. To ensure the quality of the downstream analyses, mitochondrial genes, ribosomal protein genes, and TCR/BCR genes were removed from HVGs. The ScaleData function was then used with the parameter “vars.to.regress = “percent.mt”” to regress out the effects of the percentage of mitochondrial gene count.

To reduce noise, a principal component analysis (PCA) matrix was calculated using RunPCA with default parameters. Batch effects from different samples were removed using Harmony (version 1.0) immediately after the PCA step. Uniform Manifold Approximation and Projection (UMAP) and clustering were performed on the “Harmony space” to identify clusters. The main immune cell types were annotated based on the expression pattern of DEGs and well‐known cellular markers from the literature.

During the first round of unsupervised clustering of all cells, we observed co‐expression of PTPRC and HBB in some clusters. Therefore, these clusters were removed from the dataset for downstream analysis.

To identify sub‐clusters within BMSCs, a second round of unsupervised clustering was conducted on BMSCs cells. The second‐round clustering process, which was similar to the first round, commenced with the expression matrix of the subset of the major cell types. Next, HVGs were identified, PCA matrix was computed, and batch effects were preprocessed with Harmony. For cluster detection, the Louvain algorithm was employed, and a dimensionality reduction was conducted for visualization. The number of principal components in each major subtype was determined independently by the Elbowplot function in Seurat. Marker genes were detected using the FindAllMarkers function with standard parameters.

### Differential Expression Analysis

To identify DEGs between two clusters, the FindMarkers function was utilized to conduct a differential gene expression analysis. Genes with an adjusted P‐value of less than 0.05 were deemed as significant and differentially expressed.

### Luciferase Reporter Assay

The promoter region selected for analysis was the −2000 — ‐1 bp of the *Usp26* encoding region. This promoter region was then analyzed using the JASPAR core database, which identified the presence of five putative binding sites for Hif‐1α at −1 — −500 bp (−191 — −198 (2 putative binding sites on both sense strand and antisense strand), −262 — −269, −450 — −457, −437 — −444), two putative binding sites for Hif‐1α at −500 — −1000 bp (−626 — −633, −962 — −969), and 5 putative binding sites for Hif‐1α at −1000 — −1500 bp (−1128 — −1135, −1140 — −1147, −1174 — −1181, −1262 — −1269, −1264 — −1271) in the promoter region of Usp26. To further investigate which predicted binding site is essential for Usp26 promoter regulation by HIF‐1α, −1 → 500 bp, −500 → 1000 bp, and −1000 → 1500 bp promoter regions of the mouse Usp26 were constructed. The specific sequence for these HREs was RCGTG, where R is either A or G. The *Usp26* promoter reporter was directly cloned into a pGL3‐Basic luciferase vector using the primers in Table S4 (Supporting Information) to amplify the mouse *Usp26* promoters by PCR. Mouse primary BMSCs were then seeded into 24‐well plates and co‐transfected with different plasmids, including firefly reporter constructs containing the *Usp26* promoter, a Renilla‐expressing plasmid, and varying doses of HIF‐1α plasmid. Firefly and Renilla luciferase activities were measured 24 h post‐transfection using a Dual Luciferase Assay System (Promega).

### Hypoxic Culture of BMSCs

Cells from bone marrow were flushed with α‐MEM through the femur and tibia with 1 mL injection syringe. These cells were expanded using the mouse MesenCult Expansion Kit (STEMCELL Technologies) following the manufacturer's protocols.^[^ ^60^ ^]^ Then the cells were incubated in a hypoxic incubator (2% oxygen) for 24 h for subsequent experiments.^[^ ^66^ ^]^

### Usp26 Overexpression in Aged BMSCs

The Usp26 gene was cloned into a lentiviral vector backbone‐pLV[Exp]‐EGFP:T2A:Puro‐EF1A using the Golden Gate method, and mCherry was inserted into the same vector as a negative control.^[^ ^9^ ^]^ Young BMSCs collected from 2‐month‐old mice and O‐BMSCs collected from 20‐month‐old mice were transfected with either Usp26 overexpression lentivirus or control lentivirus. Specifically, young BMSCs transfected with control lentivirus (Y‐BMSCs‐Ctrl), O‐BMSCs transfected with control lentivirus (O‐BMSCs‐Ctrl), and O‐BMSCs transfected with Usp26 overexpression lentivirus (O‐BMSCs‐U). 48 h post‐transfection, flow cytometry was used to obtain 1 × 10^5^ Y‐BMSCs‐Ctrl (CD44^+^ CD105^+^mCherry^+^), O‐BMSCs‐Ctrl (CD44^+^ CD105^+^mCherry^+^), and O‐BMSCs‐U (CD44^+^ CD105^+^ EGFP^+^) for further evaluation of self‐renewal and differentiation potential of BMSCs over multiple passages.

*ChIP‐qPCR*: The ChIP procedure was performed as previously described,^[^ ^67^ ^]^ utilizing the ChIP assay kit (Millipore Sigma, Burlington, MA). Briefly, the cells were fixed with formaldehyde, and 125 mM glycine was used to neutralize them. Lysing of the cells was accomplished with a mixture of 5 mM PIPES pH 8, 85 mM KCl, 0.5% NP‐40, 20 mM sodium butyrate, and protease/phosphatase inhibitors (2 mM PMSF, 20 mM NaF, 1 × Aprotinin, 0.1 mg mL⁻^1^ Leupeptin, 2 mM Na3VO4). Chromatin fragments of 100–500 base pairs were obtained through sonication with the Bioruptor sonicator (Diagenode, Denville, NJ). Using 3 µg of HIF‐1α antibody (NOVUS, NB100‐105), or negative control anti‐IgG (CST, #5415), fragmented chromatin (200 µg) was incubated overnight to form immune‐complexes, which were coupled to Protein A beads. Crosslinks were reversed (65 °C for 12–16 h), and then the precipitated DNA was purified using the QIAquick PCR purification kit (Qiagen, Valencia, CA, USA) after treatment with Proteinase K and RNase A. The input and purified HIF‐1α‐ChIP‐DNA were used in qPCR. The values obtained from the immunoprecipitated samples were normalized to those from the input DNA. The DNA‐star Lasergene 15.2 core suite (DNA‐star, Madison, WI) was used to design primers for the putative HIF‐1α‐binding regions, −191 — ‐198 bp, −262 — ‐269 bp and −450 — −457 & −437 — ‐444 bp on the promoters of *Usp26*. Between −191 and ‐198 bp, there are two HIF‐1α binding sites. However, since they are found on both the sense and antisense strands, a set of primers were created. Additionally, because of the proximity of −450 to ‐457 bp and −437 to ‐444 bp, it was unable to create primers for these regions separately. As a result, a set of primers that cover both the −450 to ‐457 bp and −437 to ‐444 bp regions were developed. All primers were designed through Primer‐BLAST, and the primer sequences were included in Table S5 (Supporting Information). Immunoprecipitated DNA was analyzed through RT‐qPCR as previously described. Using the ^ΔΔ^Ct method and normalizing to % input, fold changes were calculated and presented as fold enrichment over the control mouse IgG.

### Adenovirus Infection

BMSCs were cultured in 24‐well plate at a density of 4 × 10^5^‐5 × 10^5^ per well at 37 °C, 5% CO_2_ overnight. First, the adenovirus was placed on the ice and slowly dissolved. Half of the fresh serum‐free medium and half of the adenovirus with GFP (Genepharma, Chian) were added to the dish. Blank adenovirus containing GFP was added as control. After 6 h of infection, the medium was replaced with a fresh complete medium. After 12–16 h of infection, the culture medium containing the virus was removed and a fresh complete culture medium was replaced. Fluorescence microscopy (ZEISS, Germany) was used to detect the infection efficiency of GFP 36–48 h after infection.

### Quantification and Statistical Analysis

Statistical analyses were performed using GraphPad Prism 9. Statistical methods are graphically illustrated in the legend. Statistical significance was determined by two‐sided Student's t test when comparing the differences between two groups. One‐ or two‐way ANOVA was used to compare the differences among more than two groups. For all statistical tests, we considered P value < 0.05 to be statistically significant.

## Conflict of Interest

The authors declare no conflict of interest.

## Author Contributions

Y.X., L.C., and Y.C. contributed equally to this work. C.L. and L.D conceived and designed the experiments; Y.X., L.C., Z.D., L.Z., and J.T. performed the experiments; C.L. and Y.X. analyzed the data; C.L., Y. C., G. T., and L.D. contributed reagents/materials/analysis tools; C.L. wrote, review and revision of the manuscript. All authors read and approved the final paper. The schematic diagrams (Figure 2A, Figure 3A, Figure 3L, Figure 4A, Figure 5A, Figure 5P, Figure 7A, Figure 7M, Figure 9A, Figure 11; Figure S4A, S5A, Supporting Information) and graphic abstract were created with BioRender.com.

## Supporting information



Supporting Information

Supporting Information file 1

Supporting Information file 2

Supporting Information file 3

Supporting Information file 4

## Data Availability

Research data are not shared.
