# Supplementary material for: USP26 Combats Age‐Related Declines in Self‐Renewal and Multipotent Differentiation of BMSC by Maintaining Mitochondrial Homeostasis
Source: Adv Sci (Weinh). 2024 Oct 8;11(44):2406428. doi: 10.1002/advs.202406428 (PMC11600297; doi:10.1002/advs.202406428)
Supplement: Supplementary file 1 — Supporting Information [file ADVS-11-2406428-s002.docx]

**Supplementary Materials for**

**USP26 combats age-related declines in self-renewal and multipotent differentiation of BMSC by maintaining mitochondrial homeostasis**

*Yiming Xu^1#^, Leilei Chang^1#^, Yong Chen^2,3#^, Zhou Dan^1^, Li Zhou^2,3^, Jiyuan Tang^1^, Lianfu Deng^1^*, Guoqing Tang^2,3^*, Changwei Li^1^**

^1^ Department of Orthopedics, Shanghai Key Laboratory for Prevention and Treatment of Bone and Joint Diseases, Shanghai Institute of Traumatology and Orthopedics, Ruijin Hospital, Shanghai Jiaotong University School of Medicine, 197 Ruijin 2nd Road, Shanghai, 200025, China.

^2^ Department of Orthopedics, Kunshan Hospital of Chinese Medicine, Affiliated Hospital of Yangzhou University, Suzhou, Jiangsu Province, 215300, China.

^3^ Institute of Traumatology and Orthopedics, Kunshan Hospital of Chinese Medicine, Affiliated Hospital of Yangzhou University, Suzhou, Jiangsu Province, 215300, China.

^#^ These authors contributed equally to this work.

* Corresponding authors:

Email: lcw11876@rjh.com.cn (Changwei Li); 17712485172@yzu.edu.cn (G. Tang); lfdeng@shsmu.edu.cn (L. Deng)

**This PDF file includes:**

Figures S1 to S11

Tables S1 to S5


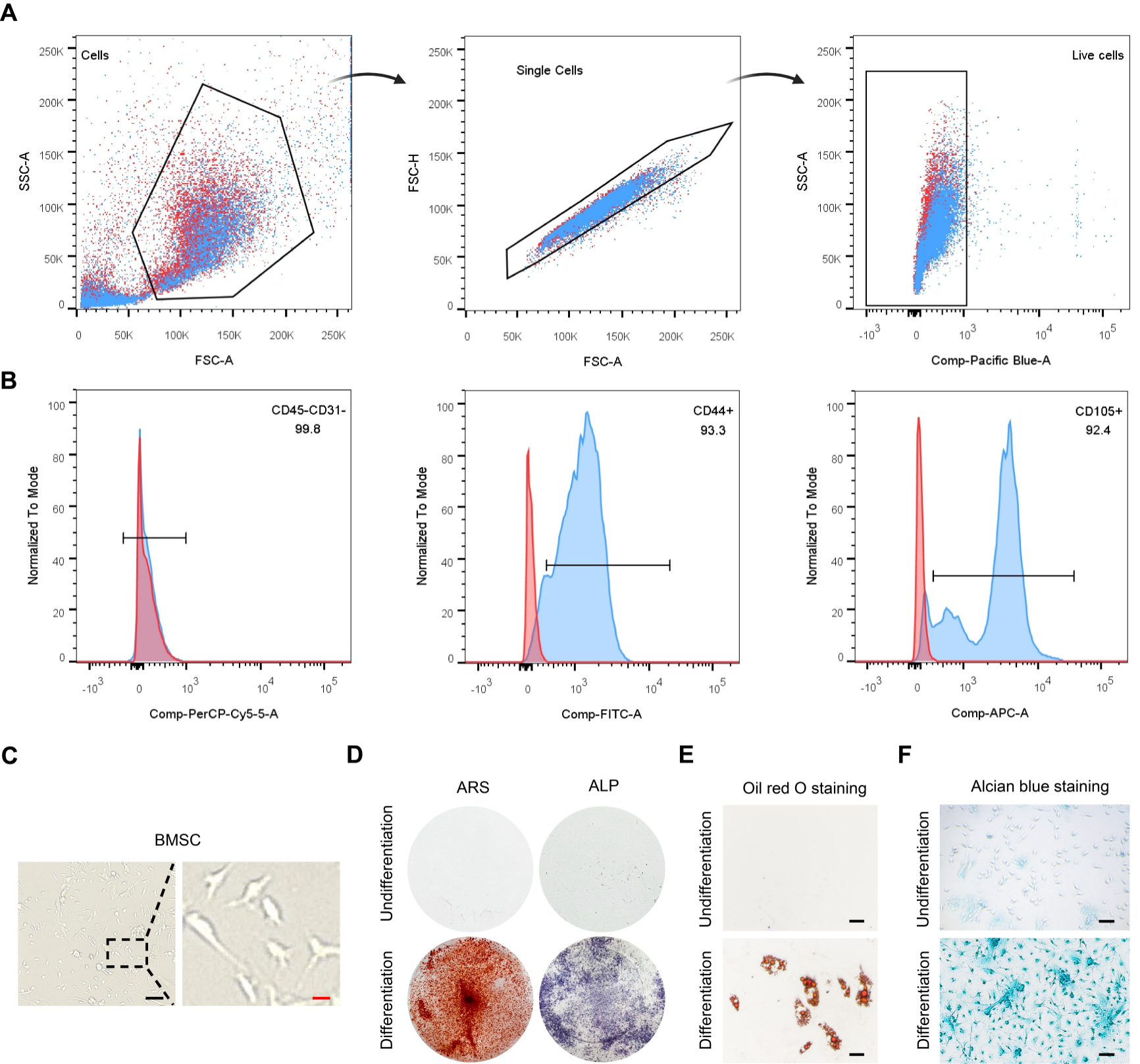


**Figure S1**. The identification of BMSCs. A) The bone marrow cells were flushed from the femur and tibia using a 1ml injection syringe filled with α-MEM. P3 cells were stained with specific antibodies for CD45, CD31, CD105, and CD44; FACS analysis was performed, and living cell populations were gated using DAPI. B) Within the gate of living cells, most of the cells were identified as CD45^-^CD31^-^ (99.8%), CD44^+^ (93.3%), and CD105^+^ (92.4%). C) Light microscope images of BMSCs. Scale bar, 20 μm (black) and 2.5 μm (red). D) Representative images of ARS and ALP staining of BMSCs after 14 days of osteogenic differentiation. n=6 in each group. E) Representative images of Oil red O staining of BMSCs after 21 days of adipogenic differentiation. n=6 in each group. Scale bar, 5 μm (black). F) Representative images of alcian blue staining of BMSCs after 7 days of chondrogenic differentiation. n=6 in each group. Scale bar, 25 μm.


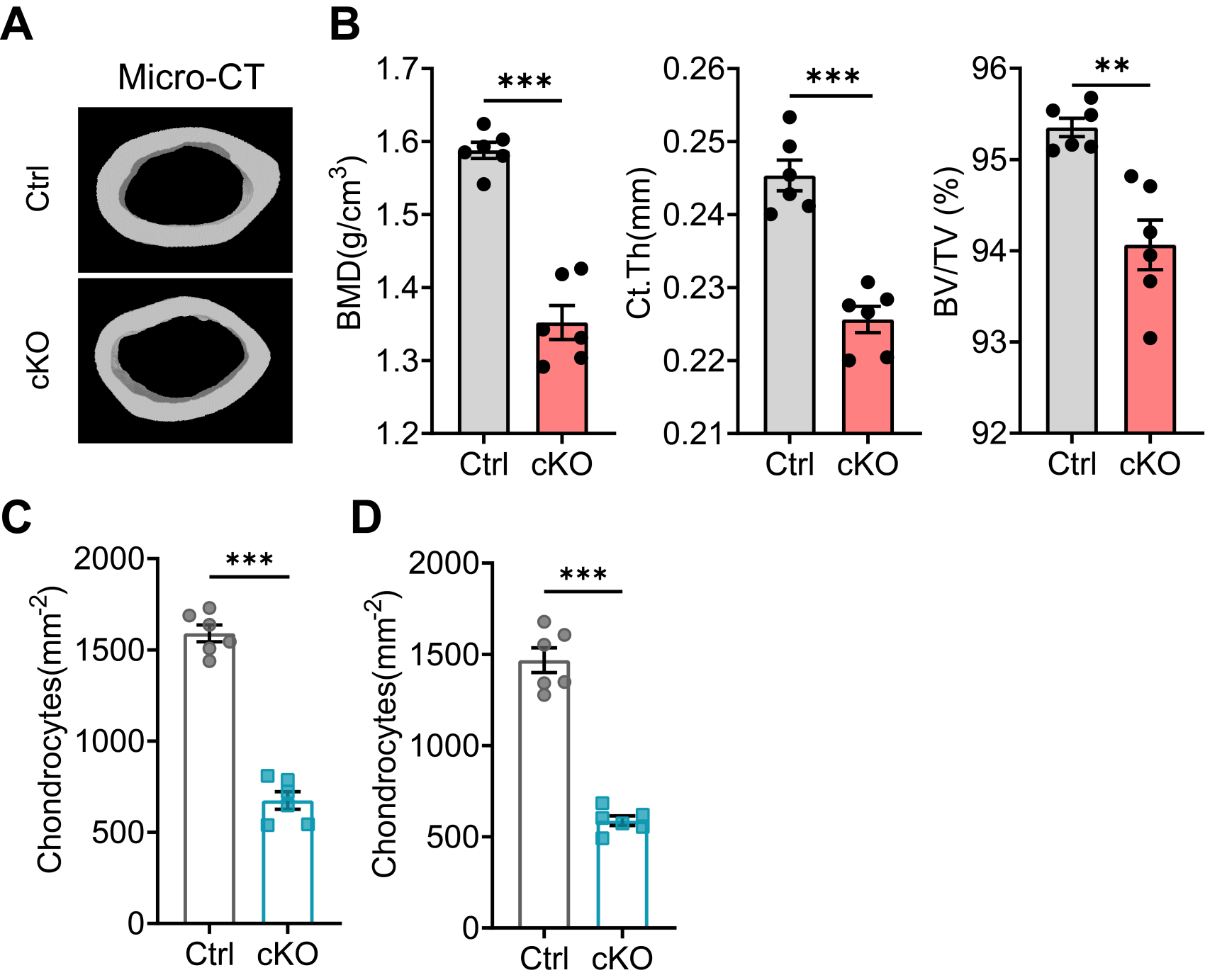


**Figure S2.** A) Representative Micro-CT images of cortical bone of the femoral metaphysis from control and cKO mice. n=6 in each group. B) Quantitative analysis of the cortical bone including BMD, Ct.Th, and BV/TV. n=6 in each group. C) The number of chondrocytes per tissue area was measured based on H&E staining in Figure 2T. n=6 in each group. D) The number of chondrocytes per tissue area was measured based on Alcian blue staining in Figure 2U. n=6 in each group. Mice age in A) and B), 2-month-old. Mice age in C) and D), E16.5. Data are represented as mean ± SD. Statistical significance was determined by two-sided student’s t test. ***p* < 0.01, ****p* < 0.001.


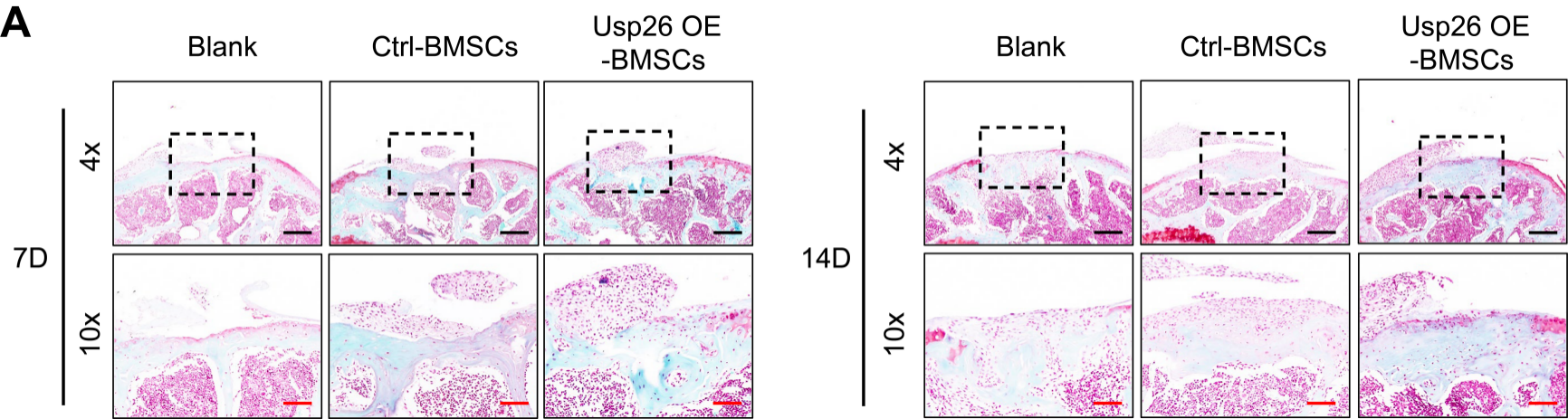


**Figure S3**. A) Representative Safranin O/Fast green staining images of femoral osteoarticular tissues from different groups are shown. n=6 in each group. The scale bar is indicated as 200 μm (black) and 100 μm (red). Mice age, 8-week-old.


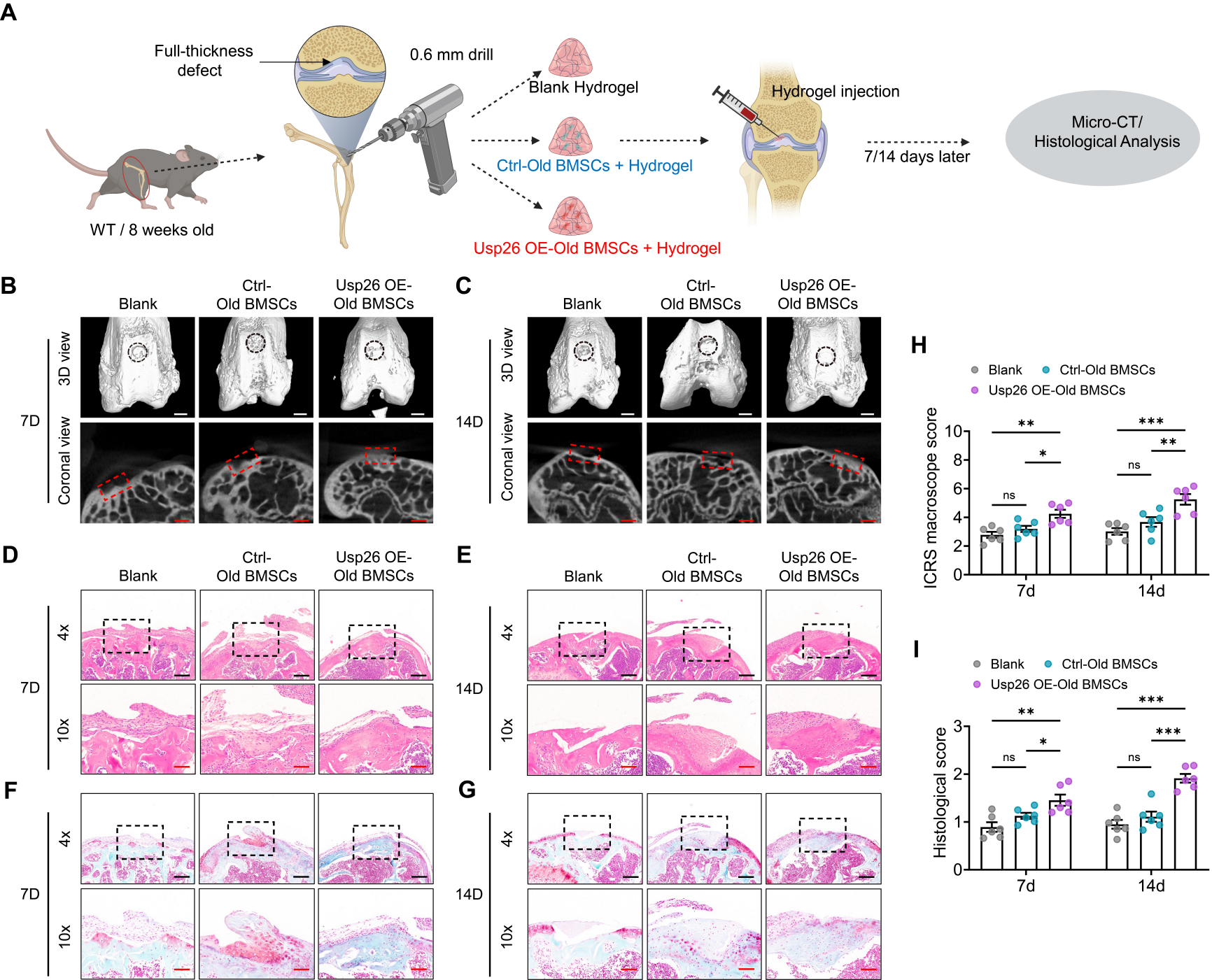


**Figure S4.** Usp26 enhanced the therapeutic cartilage repair function of senescent BMSCs. A) 0.6 mm holes were generated in femoral bones of 8-week-old WT male mice. The defects did not penetrate the subchondral bone, and were filled with blank hydrogel, control-Old BMSCs + hydrogel, or Usp26 OE (overexpression)-Old BMSCs + hydrogel. After the surgery, defect bone samples were collected for Micro-CT scanning and histological analysis. B-C). Micro-CT images and coronal views of the femoral osteoarticular bone from different groups at 7 and 14 days after surgery. n=6 in each group. Scale bar, 500 μm (white) and 300 μm (red). D-E). Representative H&E staining images of femoral osteoarticular bone from different groups. n=6 in each group. Scale bar, 200 μm (black) and 100 μm (red). F-G) Representative Safranin O/Fast green staining images of femoral osteoarticular bone from different groups. n=6 in each group. Scale bar, 200 μm (black) and 100 μm (red). H) Analysis of ICRS macroscope score for the harvested samples. n=6 in each group. I) Histological score for the harvested samples. n=6 in each group. Mice age in A) to I), 8-week-old. BMSCs from mice at P5 were used in A). Data are represented as mean ± SD. Statistical significance was determined using one-way ANOVA. **p* < 0.05, ***p* < 0.01, ****p* < 0.001.


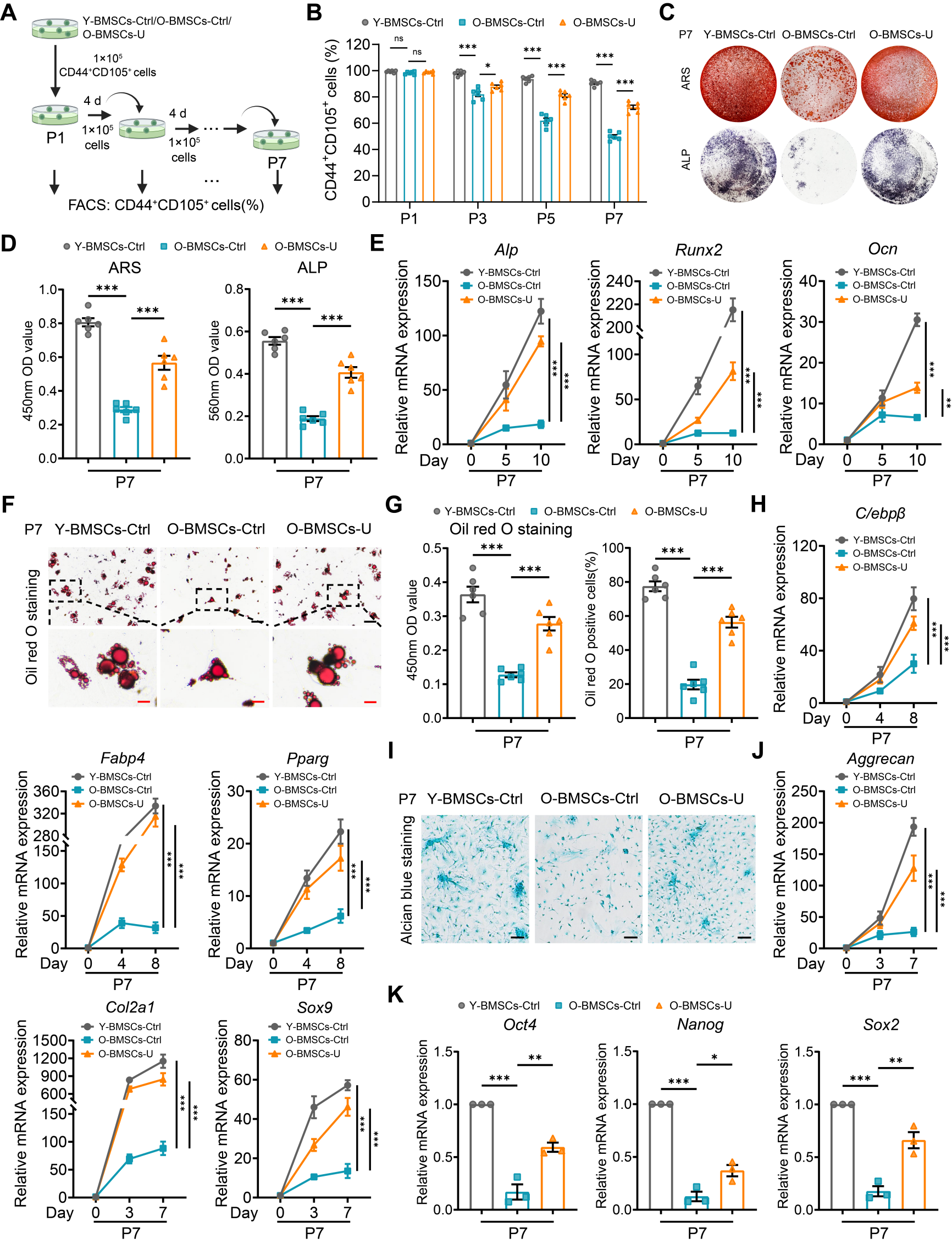


**Figure S5.** Supplementation of Usp26 reverses the impaired self-renewal and multipotent differentiation of Old BMSCs. A) Schematic diagram illustrating the FACS analysis of BMSCs during serial passaging. B) The percentage of CD44 and CD105 positive cells in BMSCs at different passages. n=6 in each group. C) Representative images of ARS and ALP from specific groups after 14 days of osteogenic induction. n=6 in each group. D) Statistical analysis of the absorbance at 450 nm of ARS and the absorbance at 560 nm of ALP staining. n=6 in each group. E) qPCR analysis of Alp, Runx2, and Ocn mRNA expressions in BMSCs from specific groups after different days (0, 5, and 10 days) of osteogenic induction. n=3 in each group. F) Representative images of Oil red O staining from specific groups after 21 days of adipogenic differentiation. n=6 in each group. Scale bar, 20 μm (black) and 5 μm (red). G) Statistical analysis of the absorbance at 450 nm of Oil red O staining and percentage of Oil red O positive cells from specific groups. n=6 in each group. H) qPCR analysis of C/ebpβ, Fabp4, and Pparg mRNA expressions in BMSCs from specific groups after different days (0, 4, and 8 days) of adipogenic differentiation. n=3 in each group. I) Representative images of alcian blue staining of BMSCs from specific groups after 7 days of chondrogenic differentiation. n=6 in each group. Scale bar, 25 μm. J) qPCR analysis of Aggrecan, Col2a1, and Sox9 mRNA expressions in BMSCs from specific groups after different days (0, 3, and 7 days) of chondrogenic differentiation. n=3 in each group. K) qPCR analysis of Oct4, Nanog, and Sox2 in P7 Y-BMSCs-Ctrl, O-BMSCs-Ctrl and O-BMSCs-U. n=3 in each group. Young mice age, 2-month-old. Old mice age, 20-month-old. BMSCs from mice at P7 were used in C)-K). Data are represented as mean ± SD. Statistical significance was determined by one-way ANOVA in D), G), and K), or two-way ANOVA in B), E), H) and J). **p* < 0.05, ***p* < 0.01, ****p* < 0.001.


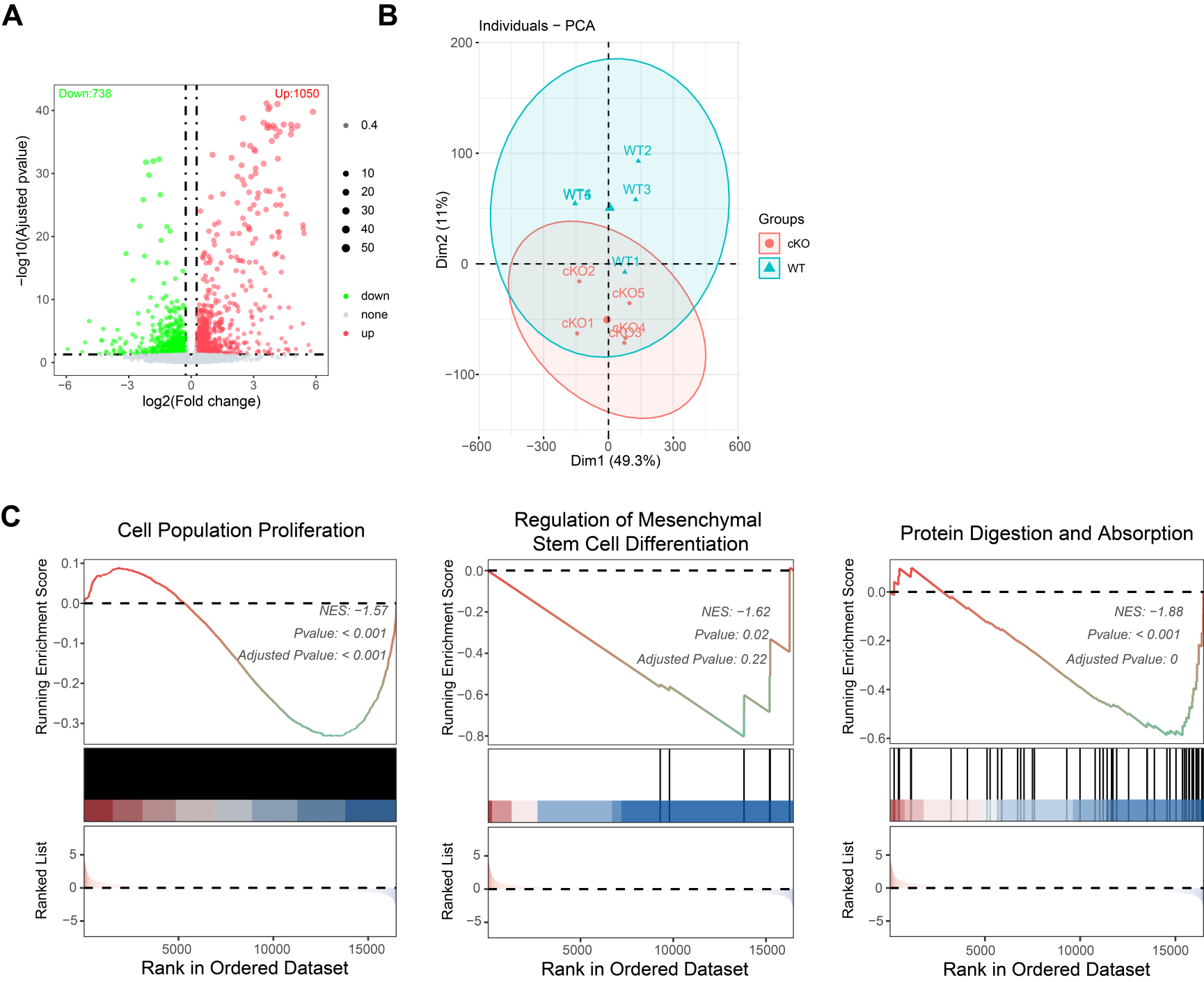


**Figure S6.** RNA-Seq analysis showed that decrease of Usp26 caused significant differentially expressed mRNA in BMSCs. A) Volcano plots showing differentially expressed genes between BMSCs from control and cKO mice. The green and red dots represent the down-regulated and up-regulated genes, respectively. B) Principal component analysis (PCA) showing differentially expressed genes between BMSCs from WT (WT1-5) and cKO (cKO1-5) mice. C) GSEA showing significant differentially enrichment of genes in the pathways of cell population proliferation, regulation of mesenchymal stem cell differentiation, and protein digestion and absorption.


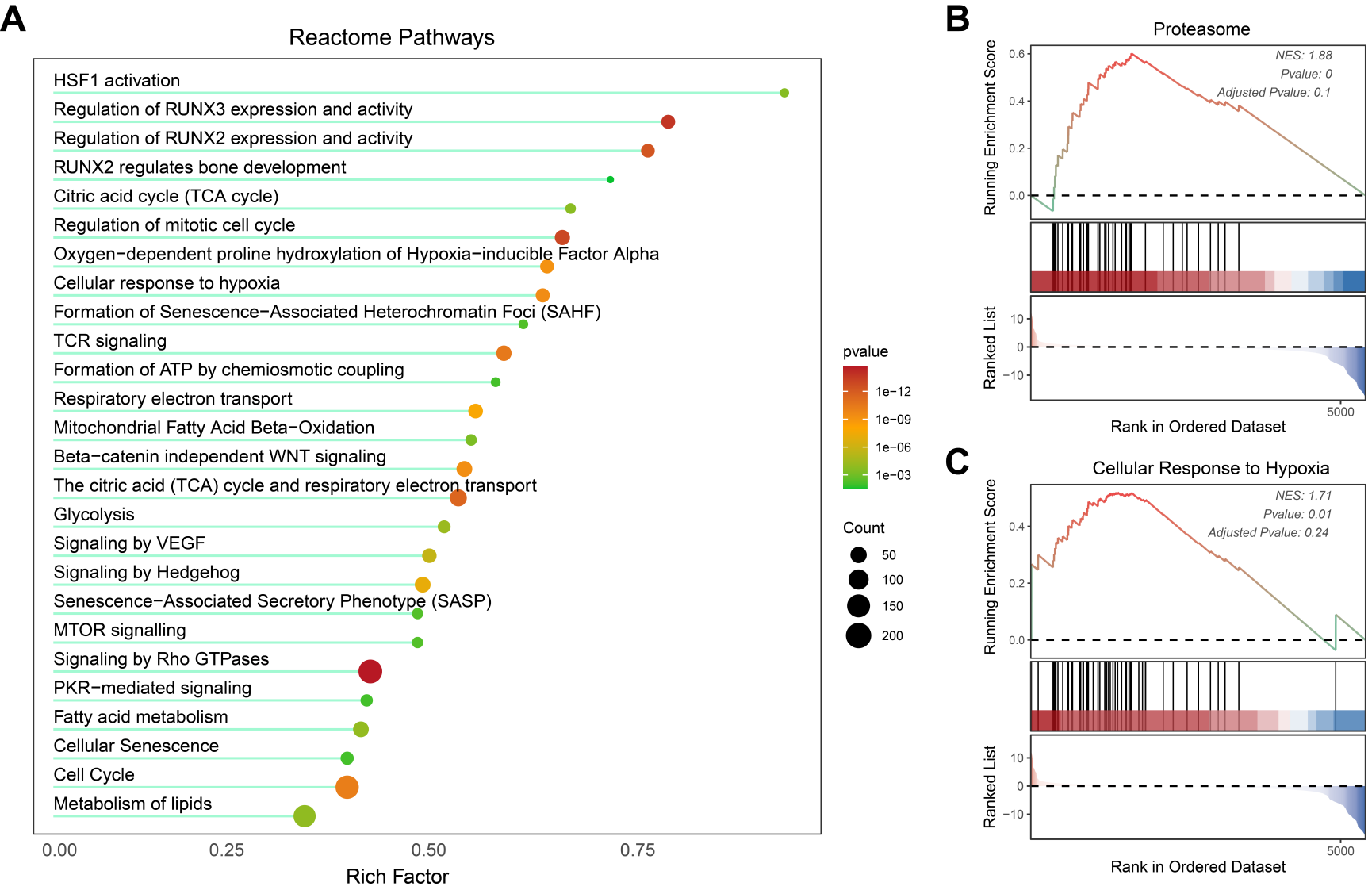


**Figure S7.** Proteomics analysis showed that decrease of Usp26 resulted in significant differentially expressed proteins in BMSCs. A) The bubble chart of Reactome analysis for differentially expressed pathways between BMSCs from control and cKO mice. B) GSEA showing significant differentially enrichment of proteins in the pathways of Proteasome. C) GSEA showing significant differentially enrichment of proteins in the pathways of Cellular Response to Hypoxia.





**Figure S8.** USP26 regulated aging, self-renewal and multipotency through SIRT2 in BMSCs. A) Representative EdU staining images and percentage of EdU positive cells in control BMSCs, cKO BMSCs, cKO BMSCs treated with Usp26 overexpression lentivirus, and with or without shSirt2 lentivirus were shown. n=6 in each group. Scale bar, 20 μm. B) Representative images of CFU in BMSCs from specific groups. The colony number serves as a quantitative measurement. n=6 in each group. C) Western-blot analysis was conducted to assess P16, P21, OCT4, NANOG, SOX2, MFN2, FIS1, and SIRT2 protein levels in BMSCs from each group. n=3 in each group. D-E) Representative SA-β-Gal staining images and the percentage of SA-β-Gal positive cells in specific groups are presented. n=6 in each group. Scale bar, 25 μm. F) qPCR analysis was performed to evaluate P16 and P21 mRNA expressions in BMSCs from each group. n=3 in each group. G) qPCR analysis was carried out to examine Oct4, Nanog, and Sox2 mRNA expressions in BMSCs from each group. n=3 in each group. Mice were 8-weeks-old. Mice age in A) to G), 2-month-old. BMSCs from mice at P5 were used in A)-G). Data are represented as mean ± SD, and statistical significance was determined by one-way ANOVA. **p* < 0.05, ***p* < 0.01, ****p* < 0.001.


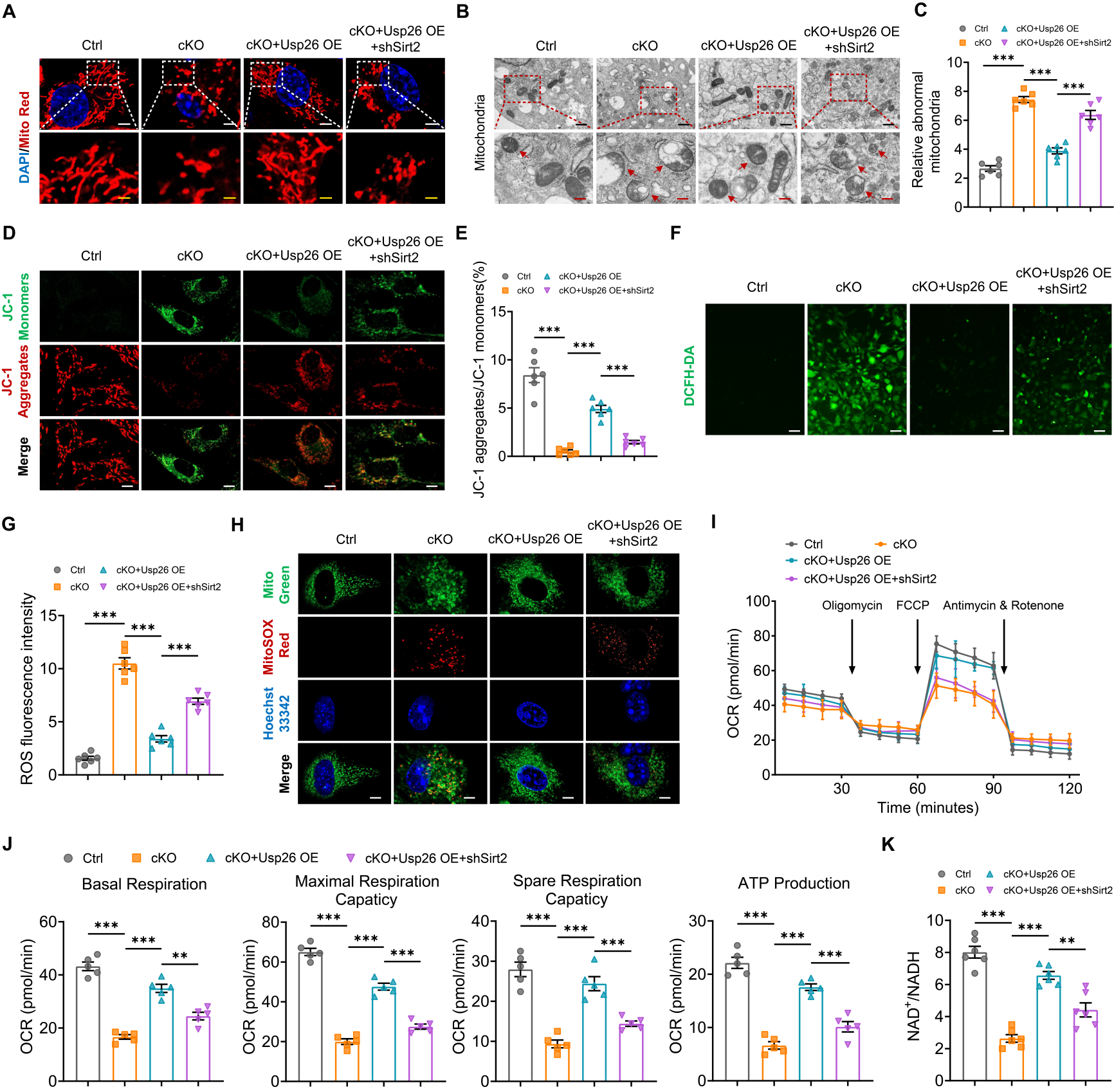


**Figure S9.** USP26 regulated mitochondrial function through SIRT2. A) Representative images of mitochondria from control BMSCs, cKO BMSCs, cKO BMSCs treated with Usp26 overexpression lentivirus, and cKO BMSCs treated with Usp26 overexpression and shSirt2 lentivirus are shown, visualized with MitoTracker Red staining. n=6 in each group. Scale bar, 2 μm (white) and 0.5 μm (yellow). B) Representative transmission electron microscopy (TEM) images of mitochondria from specific groups are shown. n=6 in each group. Scale bar, 500 nm (black) and 200 nm (red). C) Relative rates of abnormal mitochondria in BMSCs from specific groups. n=6 in each group. D) Detection of JC-1 monomers (green) and aggregates (red) by confocal fluorescence microscopy from particular groups. n=6 in each group. Scale bar, 4 μm. E) The ratio of JC-1 aggregates/JC-1 monomers in BMSCs from particular groups. n=6 in each group. F-G) Representative images of the DCFH-DA assay displaying intracellular ROS levels in BMSCs from specific groups. ROS fluorescence intensity used as a quantitative measurement. n=6 in each group. Scale bar, 15 μm. H) Representative images of mtROS from specific groups visualized with MitoSOX (red) staining. n=6 in each group. Scale bar, 2 μm. I) Detection of the oxygen consumption rates (OCR) from specific groups in response to indicated mitochondrial modulators (Oligomycin, FCCP, Antimycin & Rotenone). n=5 in each group. J) Calculation of basal respiration, maximal respiration capacity, spare respiration capacity, and ATP production of BMSCs from specific groups based on the OCR values. n=5 in each group. K) The ratio of NAD^+^/NADH from specific groups. n=6 in each group. Mice were 8-weeks-old. Mice age in A) to K), 2-month-old. BMSCs from mice at P5 were used in A)-K). Data are represented as mean ± SD. Statistical significance was determined by one-way ANOVA. ***p* < 0.01, ****p* < 0.001.


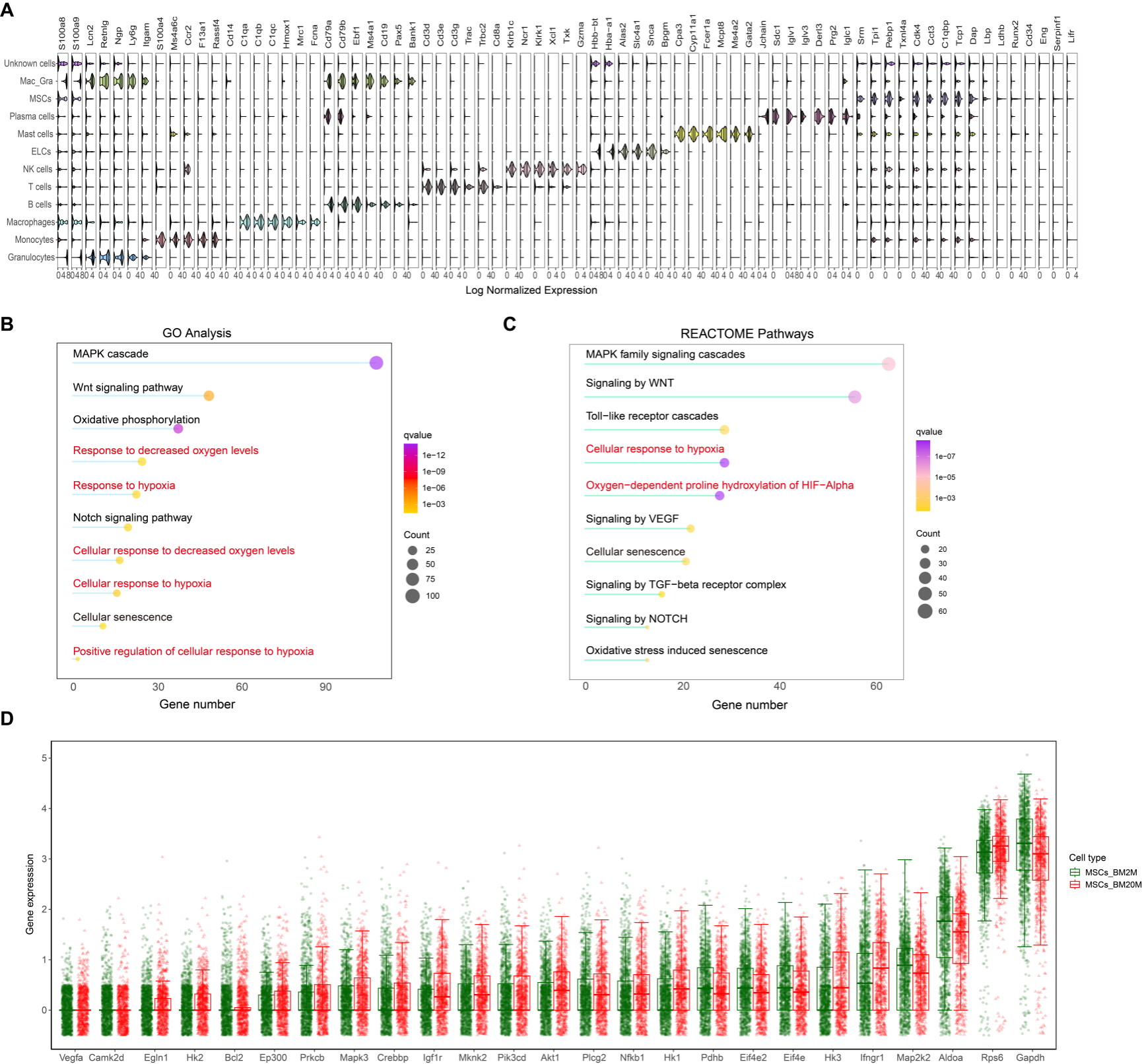


**Figure S10.** Single-cell RNA-Seq showed HIF-1α regulated the expression of USP26 through transcription. A) Violin plot of average expression of canonical marker genes for different cell types. B) The bubble chart of GO analysis for differentially expressed mRNA between BMSCs from 2-month-old and 20-month-old mice. C) The bubble chart of REACTOME Pathways analysis for differentially expressed mRNA between BMSCs from 2-month-old and 20-month-old mice. D) Difference in the expression levels of canonical marker genes regulated by HIF-1α.


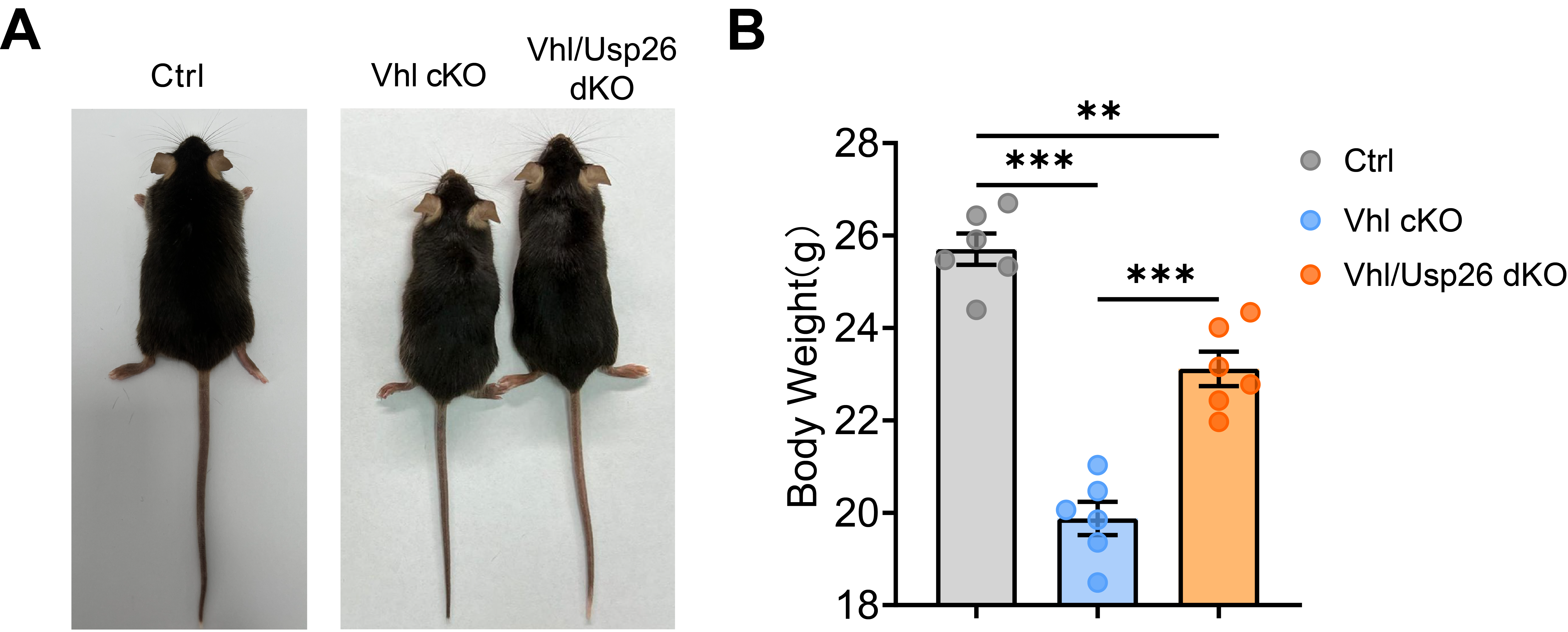


**Figure S11.** The body changes between Ctrl, Vhl cKO, and Vhl/Usp26 dKO mice. A) Body size of different mice (Ctrl, Vhl cKO, and Vhl/Usp26 dKO). B) Body weight of different mice (Ctrl, Vhl cKO, and Vhl/Usp26 dKO). n=6 each group. Mice age, 6-month-old. Data are represented as mean ± SD. Statistical significance was determined by two-sided student’s t test. ***p* < 0.01, ****p* < 0.001.

**Table S1. Genotyping primers for transgenic mouse**

| Primer name |  | Primer sequence (5’- 3’) | PCR produce size |
| --- | --- | --- | --- |
| Vhl^flox/flox^ | FORWARD | AAGAGCACGCAGCTTAGGAG | WT:307 bp |
|  | REVERSE | TTTCTGAGTCCTGGGGATTG | Targeted:500bp |
| Usp26^flox/flox^ | FORWARD | GTCAAATTGCAGAGCTGGCTAC | WT:463 bp |
|  | REVERSE | CCCAAACTCCATTCTTAAGCACTG | Targeted:520bp |
| Prx1-Cre | FORWARD | GCGGTCTGGCAGTAA AAACTATC | WT:- |
|  | REVERSE | GTGAAACAGCATTGCTGTCACTT | Targeted:100bp |

**Table S2. Primer sequences for real-time PCR**

| Primer name |  | Primer sequence (5’- 3’) |
| --- | --- | --- |
| Nanog | FORWARD | GAACTCTCCAACATCCTGAACCTC |
|  | REVERSE | CCTTCTGCGTCACACCATTGC |
| Sox2 | FORWARD | GGTTACCTCTTCCTCCCACTCCAG |
|  | REVERSE | TCACATGTGCGACAGGGGCAG |
| Oct4 | FORWARD | CTTGCTGCAGAAGTGGGTGGAGGAA |
|  | REVERSE | CTGCAGTGTGGGTTTCGGGCA |
| P21(Cdkn1a) | FORWARD  REVERSE | TTGCCAGCAGAATAAAAGGTG  TTTGCTCCTGTGCGGAAC |
| P16(Cdkn2a) | FORWARD  REVERSE | AATCTCCGCGAGGAAAGC  GTCTGCAGCGGACTCCAT |
| Usp26 | FORWARD  REVERSE | GAGGAAGAGCATAGACCCAGTG  TGGACGGCTTTGAGTAAGTGCC |
| Alp | FORWARD  REVERSE | CCAGAAAGACACCTTGACTGTGG  TCTTGTCCGTGTCGCTCACCAT |
| Runx2 | FORWARD  REVERSE | CCTGAACTCTGCACCAAGTCCT  TCATCTGGCTCAGATAGGAGGG |
| Ocn | FORWARD  REVERSE | GCAATAAGGTAGTGAACAGACTCC  CCATAGATGCGTTTGTAGGCGG |
| C/ebpβ | FORWARD  REVERSE | CAACCTGGAGACGCAGCACAAG  GCTTGAACAAGTTCCGCAGGGT |
| Fabp4 | FORWARD  REVERSE | GGAAGCTTGTCTCCAGTGAAAAC  TGACCAAATCCCCATTTACGC |
| Pparg | FORWARD  REVERSE | TGACCTGAAGCTCCAAGAATAC  CTGTTGTAGAGCTGGGTCTTT |
| Aggrecan | FORWARD  REVERSE | CAGGCTATGAGCAGTGTGATGC  GCTGCTGTCTTTGTCACCCACA |
| Col2a1 | FORWARD  REVERSE | GCTGGTGAAGAAGGCAAACGAG  CCATCTTGACCTGGGAATCCAC |
| Sox9 | FORWARD  REVERSE | TCCTAGAACATTCACTGTGCC  GGCAACCAGGGAAAATGTG |
| IL-1α | FORWARD  REVERSE | TTGGTTAAATGACCTGCAACA  GAGCGCTCACGAACAGTTG |
| IL-1β | FORWARD  REVERSE | AGTTGACGGACCCCAAAAG  AGCTGGATGCTCTCATCAGG |
| IL6 | FORWARD  REVERSE | GCTACCAAACTGGATATAATCAGGA  CCAGGTAGCTATGGTACTCCAGAA |
| Cxcl1 | FORWARD  REVERSE | ACCCAAACCGAAGTCATAGCC  TTGTCAGAAGCCAGCGTTCA |
| Cxcl10 | FORWARD  REVERSE | GCTGCCGTCATTTTCTGC  TCTCACTGGCCCGTCATC |
| Ccl2 | FORWARD  REVERSE | CTTCTGGGCCTGCTGTTCA  CCAGCCTACTCATTGGGATCA |
| Ccl5 | FORWARD  REVERSE | GTGCTCCAATCTTGCAGTCG  AGAGCAAGCAATGACAGGGA |
| β-actin | FORWARD  REVERSE | CATTGCTGACAGGATGCAGAAGG  TGCTGGAAGGTGGACAGTGAGG |

**Table S3. Lists of antibodies**

| Antibody name | Brand | Catalog number | Dilution |
| --- | --- | --- | --- |
| OCT4 | Abcam | ab200834 | 1:10000 (WB) |
| NANOG | Abcam | ab214549 | 1:1000 (WB) |
| SOX2 | Abcam | ab92494 | 1:1000 (WB) |
| USP26 | Abcam | ab230226 | 1:1000 (WB) |
| P16 | Abcam | ab51243 | 1:1000 (WB) |
| P21 | Abcam | ab109199 | 1:1000 (WB) |
| IL-6 | Abcam | ab290735 | 1:100 (IHC) |
| HIF-1α | Abcam | ab308433 | 1:100 (IF) |
| MFN1 | Proteintech | 13798-1-AP | 1:2000 (WB) |
| MFN2 | Proteintech | 12186-1-AP | 1:5000 (WB) |
| FIS1 | Proteintech | 10956-1-AP | 1:2000 (WB) |
| SIRT1 | Proteintech | 13161-1-AP | 1:1000 (WB) |
| SIRT2 | Proteintech | 19655-1-AP | 1:5000 (WB)  1:500 (IHC) |
| SIRT3 | Cell Signaling Technology | #5490 | 1:1000 (WB) |
| SIRT4 | Cell Signaling Technology | #69786 | 1:1000 (WB) |
| SIRT5 | Cell Signaling Technology | #8779 | 1:1000 (WB) |
| SIRT6 | Cell Signaling Technology | #12486 | 1:1000 (WB) |
| SIRT7 | Cell Signaling Technology | #5360 | 1:1000 (WB) |
| β-actin | Cell Signaling Technology | #4967 | 1:1000 (WB) |

**Table S4. Primer sequences for *Usp26* promoters**

| Primer name |  | Primer sequence (5’- 3’) |
| --- | --- | --- |
| -1 — -500 | FORWARD | GCGCGGTACCTTGTTAGGTGTACAGCTATTTAGGC |
|  | REVERSE | CAAGCTTGGGCCCCGCCACAGGGGC |
| -500 — -1000 | FORWARD | GCGGTACCAACATCCCAGTACAAATCTG |
|  | REVERSE | GGCAAGCTTTTGTGCATAGGAAGTAAGGGC |
| -1000 — -1500 | FORWARD | GGCGGTACCTGGCTGTCAGGAAACTGATTTTG |
|  | REVERSE | GCGCAAGCTTTGCATTTGTTAGGTTGCAG |

**Table S5. Primer sequences for CHIP-qPCR**

| Primer name |  | Primer sequence (5’- 3’) |
| --- | --- | --- |
| -191 — -198 | FORWARD | CAGGAAAGTCCGTCCACAC |
|  | REVERSE | GATAGTTTTTTCCTTCCATTCCCC |
| -262 — -269 | FORWARD | CCCGTACAAGGCAATAATATGC |
|  | REVERSE | CAAAGTAACTCCCACGAG |
| -450—-457 & -437—-444 | FORWARD | CATTTTTCACTGTAGATCCCCG |
|  | REVERSE | CCATTGGGATGAGACTCAATTCG |
